# Structure and Mechanism of Respiratory III–IV Supercomplexes in Bioenergetic Membranes

**DOI:** 10.1021/acs.chemrev.1c00140

**Published:** 2021-06-29

**Authors:** Peter Brzezinski, Agnes Moe, Pia Ädelroth

**Affiliations:** Department of Biochemistry and Biophysics, The Arrhenius Laboratories for Natural Sciences, Stockholm University, SE-106 91 Stockholm, Sweden

## Abstract

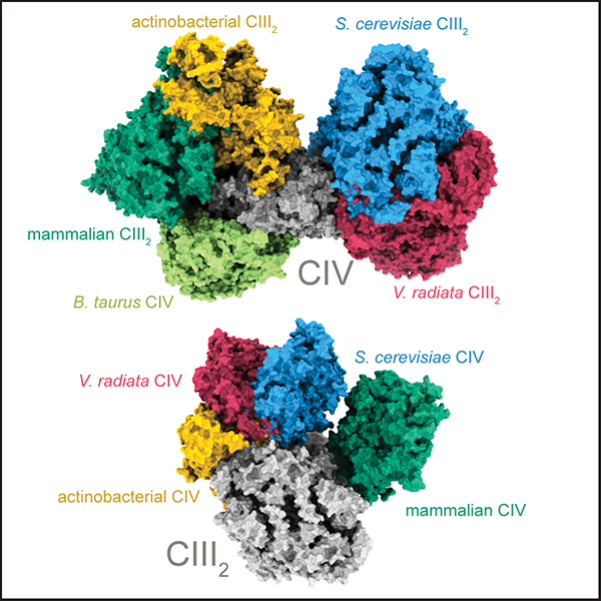

In the final steps of energy conservation in aerobic organisms, free energy from electron transfer through the respiratory chain is transduced into a proton electrochemical gradient across a membrane. In mitochondria and many bacteria, reduction of the dioxygen electron acceptor is catalyzed by cytochrome *c* oxidase (complex IV), which receives electrons from cytochrome *bc*_1_ (complex III), via membrane-bound or water-soluble cytochrome *c*. These complexes function independently, but in many organisms they associate to form supercomplexes. Here, we review the structural features and the functional significance of the nonobligate III_2_IV_1/2_*Saccharomyces cerevisiae* mitochondrial supercomplex as well as the obligate III_2_IV_2_ supercomplex from actinobacteria. The analysis is centered around the Q-cycle of complex III, proton uptake by Cyt*c*O, as well as mechanistic and structural solutions to the electronic link between complexes III and IV.

## 1 Introduction

**Figure 1:**
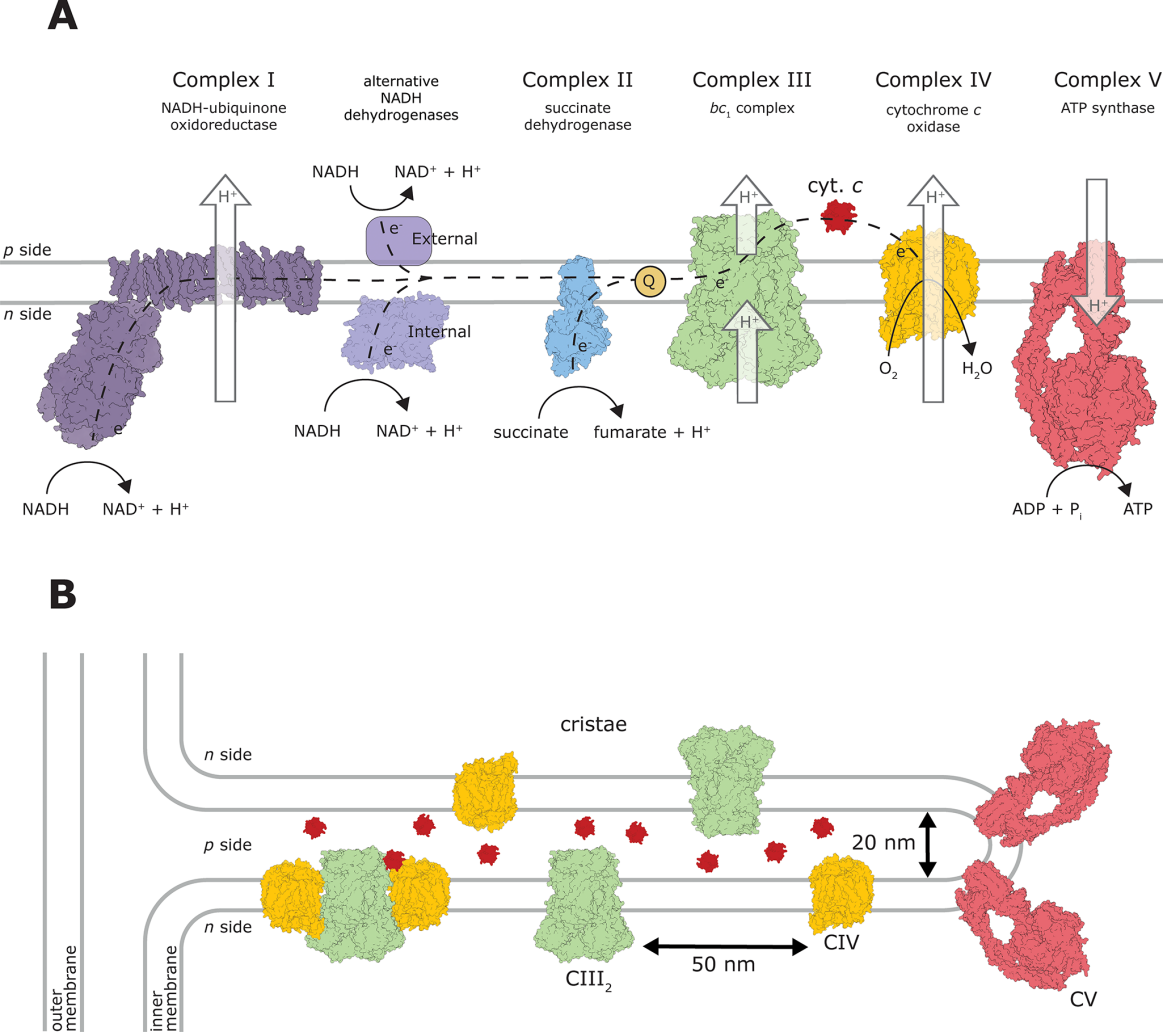
The mitochondrial respiratory chain. (A) Complex I of mammalian mitochondria is not present in *S. cerevisiae*. Instead, the external (Nde1, Nde2) and internal (Ndi1) membrane-associated NADH dehydrogenases catalyze the same NADH-oxidation:Q reduction reaction as complex I. All these enzymes are shown here in the same membrane only to illustrate the different pathways of NADH oxidation. The structures originate from different organisms: *T. thermophilus* complex I (PDB 3M9S), *S. cerevisiae* Ndi1 (PDB 4G9K), *S. scrofa* (pig) complex II (PDB 1ZOY), *S. cerevisiae* complex III and IV (PDB 6HU9), *S. cerevisiae* complex V (PDB 6CP6), and *S. cerevisiae* cyt. *c* (PDB 1YCC). (B) The respiratory chain is found in protrusions of the inner membrane that are called cristae. Here, the respiratory chain components I–IV (only complexes III and IV are shown) are located in the flat regions, while the ATP synthase (complex V) is restricted to the bent end regions. Approximate dimension and average distance are from refs (16,49−52). The cyt. *c*:Cyt*c*O ratio in *S. cerevisiae* is 2–4, which is equivalent to an average concentration of ∼100 μM cyt. *c* in the intercristae space.^16,50^

Aerobic organisms extract energy by linking oxidation of environmental compounds to production of ATP. In eukaryotes, these compounds are initially degraded to yield NADH, which is used to reduce molecular oxygen to water. Electrons from NADH are transferred through a number of enzymes that reside in the inner mitochondrial membrane. These enzymes are collectively referred to as the respiratory chain because they are wired to transfer electrons consecutively from low-potential electron donors, via a number of intermediate electron carriers, to the final, high-potential electron acceptor, O_2_. The electron current through the respiratory chain drives proton translocation across the membrane, from the inside mitochondrial matrix (negative side, *n*) to the outside intermembrane space (positive side, *p*) (Figure 1A). As a result of this process, a difference in voltage and proton concentration is maintained across the membrane, referred to as an electrochemical proton gradient or protonmotive force (PMF).^1^ The free energy that is stored in this electrochemical gradient is typically in the order ∼0.2 eV,^2,3^ and it is used for production of ATP from ADP by the ATP synthase (also known as F_1_F_o_-ATP-synthase and sometimes referred to as complex V) or for transport of molecules or ions across the membrane.^4^

In mitochondria, the energy-conversion machinery is found in protrusions of the inner membrane which define subcompartments called cristae. Here, the respiratory chain is located in the flat regions, while the ATP synthases are restricted mainly to the bent end regions^5,6^ (Figure 1B). In aerobic bacteria the respiratory chain is found in the cytoplasmic membrane where protons are translocated from the cytoplasm to the periplasm (for review, see refs (2,7−9)).

In mammalian mitochondria, the first component of the respiratory chain is an integral membrane protein called NADH:ubiquinone oxidoreductase (also named complex I), which catalyzes oxidation of NADH and reduction of quinone (Q) to quinol (QH_2_) (Figure 1A). This electron-transfer reaction is linked to pumping of protons across the membrane. Many yeast species such as *Saccharomyces* (*S*.) *cerevisiae* do not harbor a complex I, but in these mitochondria, oxidation of NADH and reduction of Q is catalyzed by other, membrane peripheral NADH dehydrogenases located both on the inner (Ndi1) and outer (Nde1 and Nde2) surfaces of the inner mitochondrial membrane^10−12^ (Figure 1A). Electron transfer to Q is also performed by succinate dehydrogenase (also named complex II). Reduced QH_2_ diffuses within the membrane to donate electrons to ubiquinol-cytochrome *c* reductase (also named cytochrome (cyt.) *bc*_1_ or complex III), which transfers electrons to water-soluble cyt. *c* that resides in the intermembrane space. Reduced cyt. *c* is an electron donor to cytochrome *c* oxidase (Cyt*c*O, also named complex IV), which catalyzes oxidation of cyt. *c* and reduction of molecular oxygen to water. Aerobic bacteria utilize a wide range of electron donors, and a specific organism may harbor many different respiratory chains that are expressed depending on environmental conditions and are often branched. General reviews of these pathways are found in refs (2,7,13−15).

Because the mobile electron carriers of the mitochondrial electron-transport chain, i.e., QH_2_ and cyt. *c*, can diffuse freely in the membrane and water phases, respectively, a functional link between the components of the respiratory chain does not require a physical linkage between these complexes. Experimental data and theoretical analyses supported a model where all respiratory complexes diffuse independently in the membrane, as do the electron carriers Q and cyt. *c*.^16^ This perception changed gradually with the invention of blue native polyacrylamide gel electrophoresis (BN-PAGE), which made it possible to identify larger complexes, referred to as respiratory supercomplexes, composed of different combinations of the respiratory enzymes with variable stoichiometry.^17^ Functionally active respiratory supercomplexes were found in a wide range of organisms.^17−28^ Recent structural studies of the inner mitochondrial membrane using electron cryo-tomography in situ demonstrated that the electron-transport chain components are organized in supercomplexes in mammals, yeast and plants,^29^ i.e., the observation of supercomplexes is not a consequence of the isolation procedures used. A wide range of these supercomplexes with different composition and stoichiometry of the components have been isolated using “weak” detergents, and in recent years a number of high-resolution supercomplex structures have been obtained using electron cryomicroscopy (cryo-EM) (reviewed in refs (30,31) and listed in Table 1).

**Table 1:** Cryo-EM Structures of Supercomplexes That Contain Complexes III_2_ and IV

composition	organism	reference	comment
III_2_IV_1_	*Vigna radiata* (mung bean)	(32)	PDB 7JRP

III_2_IV_1_ and III_2_IV_2_	*S. cerevisiae*	(33)	PDB 6T15, 6T0B
		(34)	PDB 6HU9
		(35)	PDB 6GIQ
		(36)	PDB 6YMX
		(37)	EMD 23414

I_1_III_2_IV_1_	*O. aries* (sheep)	(38)	PDB 5J4Z, 5J7Y
	*S. scrofa* (pig)	(39)	PDB 5GPN
	*S. scrofa*	(40)	PDB 5GUP
	*B. taurus* (cow)	(41)	PDB 5LUF

I_1_III_2_IV_1_ and I_2_III_2_IV_2_	*H. sapiens* (human)	(42)	PDB 5XTH, 5XTI

III_2_IV_2_	*M. smegmatis*	(43)	PDB 6ADQ
		(44)	PDB 6HWH

III_2_IV_2_	*C. glutamicum*	(45)	

III_2_IV_1_	*R. capsulatus*	(46)	PDB 6XKW, 6XKX, 6XKZ
			Cyt. *bc*_1_ and *cbb*_3_ type complex IV, including cyt. *c*_*y*_

ACIII_1_IV_1_	*F. johnsoniae*	(47)	EMD-7447
			alternative complex III from *R. marinus* also in ref ^48^

From the above discussion, it becomes apparent that the term “respiratory supercomplex” is used to describe a phenomenon, i.e., formation of membrane-bound clusters of respiratory complexes rather than entities with a well-defined composition (see Table 1). This variation in the constituents and their stoichiometry has contributed to the difficulty in uncovering a functional role of respiratory supercomplexes, which is reflected in ongoing discussions (e.g., refs (53−55)).

Many Gram-negative prokaryotes also harbor respiratory supercomplexes, but much less is known about their composition or structure (reviewed in ref (56)). For example, in *Paracoccus* (*P*.) *denitrificans*, which under aerobic conditions harbors a respiratory chain similar to that of mitochondria, supercomplexes composed of complexes III and IV were isolated already in 1985,^57^ and a larger supercomplex that included also complex I was identified later.^58^ In a recent study, a complex III–IV supercomplex from *Rhodobacter* (*R*.) *sphaeroides* that contains a membrane-anchored cyt. *c*_y_ was isolated and functionally characterized.^59^ In another recent study, the cryo-EM structure of a *Rhodobacter capsulatus* supercomplex composed of complex III, a *cbb*_3_-type complex IV and a membrane-anchored cyt. *c*_y_ was presented.^46^ Furthermore, in *Escherichia* (*E*.) *coli* cytoplasmic cell membranes a segregation of respiratory complexes into subdomains was observed *in vivo*, although these bacteria do not harbor supercomplexes.^60,61^ Gram-positive bacteria, which belong to the phylum Actinobacteria, e.g., *Mycobacterium* (*M*.) *smegmatis*, *Mycobacterium tuberculosis*, and *Corynebacterium* (*C*.) *glutamicum*, lack small *c*-cytochromes and harbor an obligate supercomplex composed of a complex III dimer flanked by two monomers of complex IV (denoted III_2_IV_2_), which are electronically linked by the diheme cyt. *cc* domain of complex III.^62−67^ A supercomplex composed of complexes III and IV was also isolated from the Gram-positive bacterium *Bacillus* PS3.^68^

**Figure 2:**
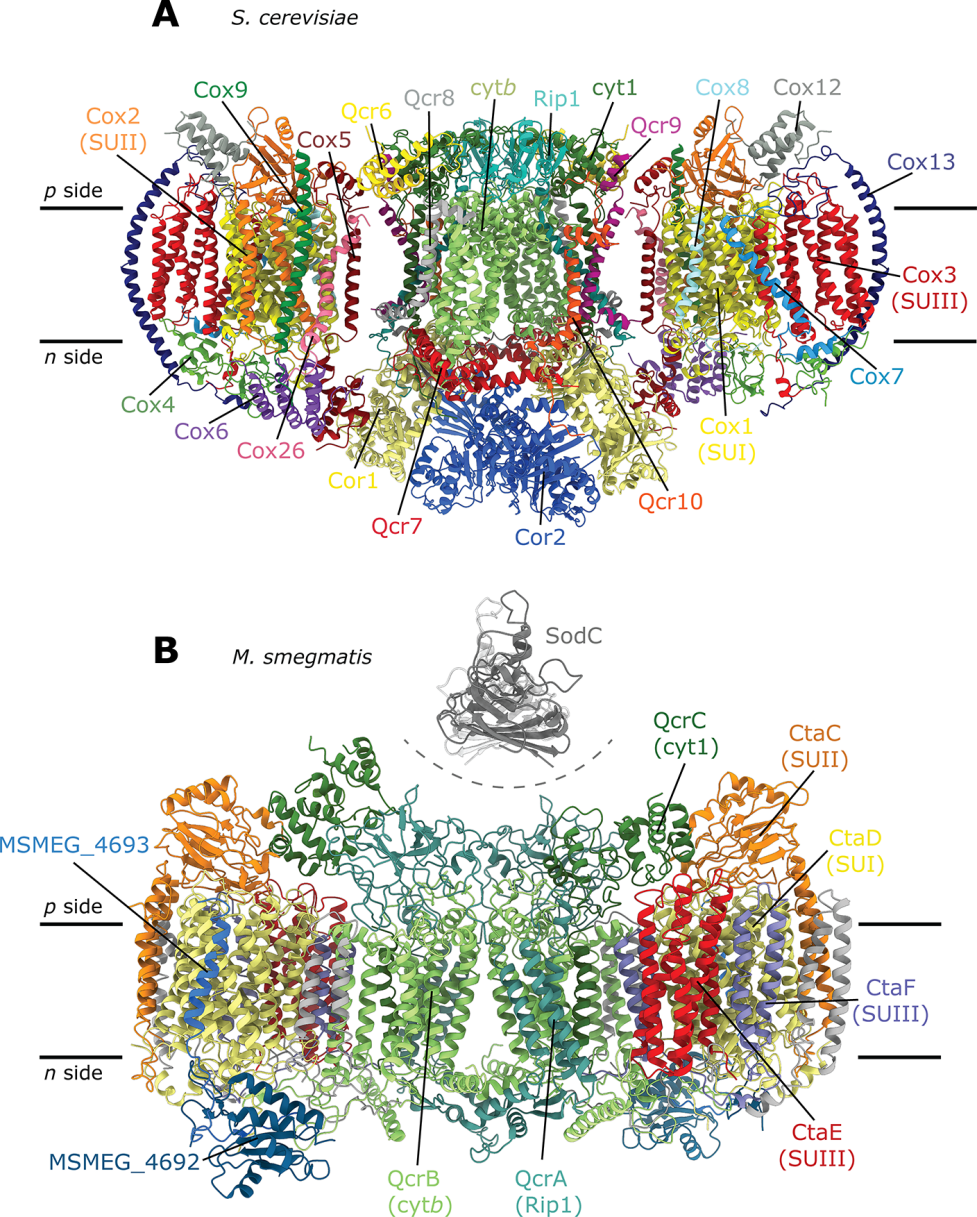
Structures of III_2_IV_2_supercomplexes. (A) *S. cerevisiae* supercomplex (PDB 6HU9). Catalytically important subunits of complexes III are cyt*b*, the Rieske iron–sulfur protein (also called Rip1 in *S. cerevisiae*) and cyt1, while those of complex IV are cox1–3 (also called SU I–III). (B) *M. smegmatis* supercomplex (PDB 6HWH, SodC is 1PZS). Catalytically important subunits of complexes III and IV are QcrA-C and CtaC-F (equivalent of SU I–III), respectively. The equivalent of canonical SU III is composed of two parts, CtaE and CtaF. Unidentified subunits are shown in gray. The SodC-type superoxide dismutase dimer subunit (PDB 1PZS) was identified in the structure.^43,44^ It was less resolved in ref (44), which did not allow identification of a connection between the subunit and the rest of the supercomplex (illustrated by the dashed line).

The *S. cerevisiae* respiratory supercomplex is composed of a cyt. *bc*_1_ dimer, flanked by either one or two Cyt*c*Os on each side of the central dimer.^17,18,69−77^ Recently determined cryo-EM structures of this supercomplex^33−35,37^ revealed its molecular architecture (Figure 2A) but also showed that the association of cyt. *bc*_1_ and Cyt*c*O does not lead to any significant structural changes of the components. This observation suggests that the functionality of the *S. cerevisiae* supercomplex is simply that of the sum of the components, except that the components reside at a fixed intercomplex distance. In contrast, structural and functional studies of the *M. smegmatis*(43,44) (Figure 2B) and *C. glutamicum*(45,62,65) supercomplexes revealed intercomplex connections that presumably modulate the functionality of the components, consistent with the obligate nature of these supercomplexes.

**Figure 3:**
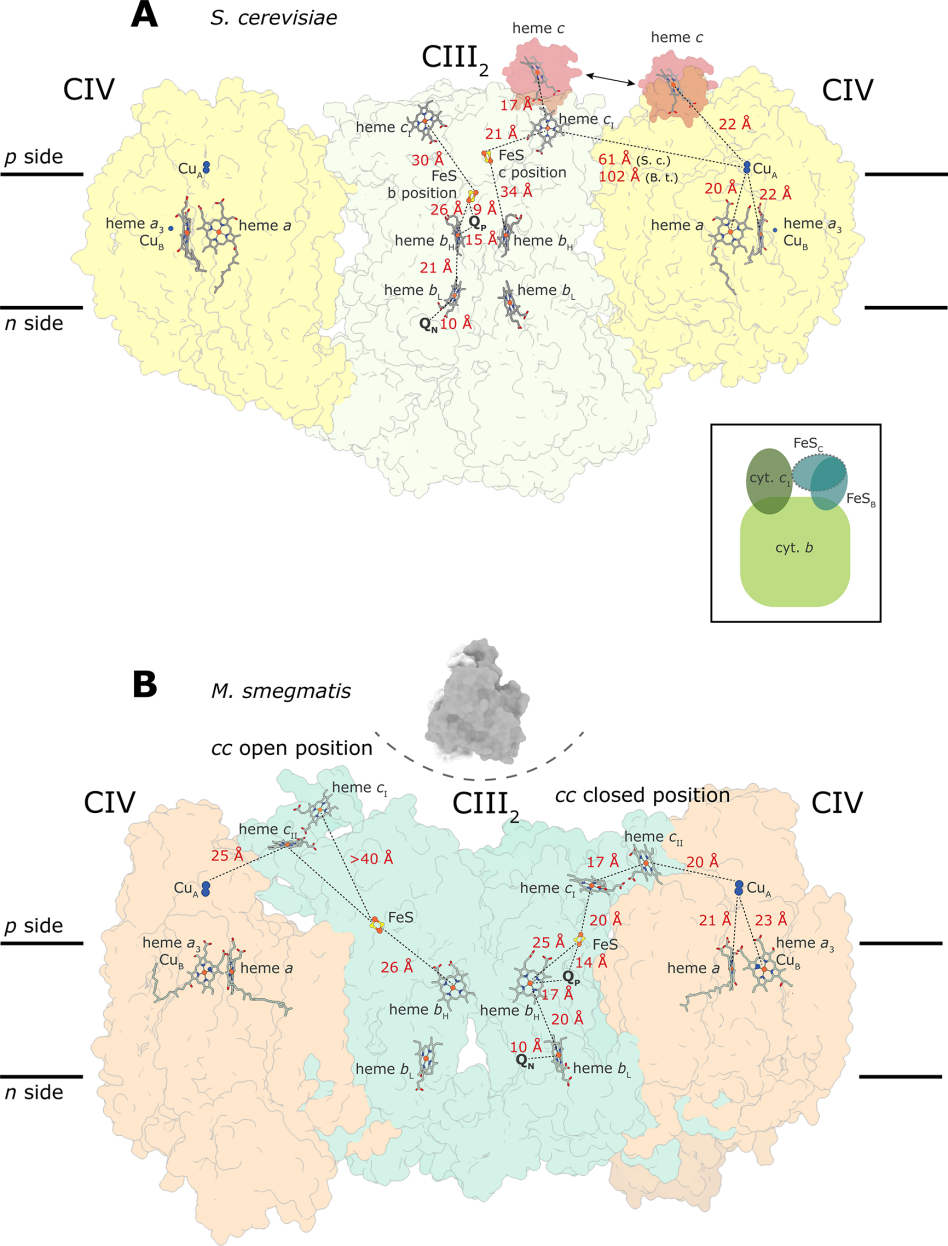
Distances between cofactors. (A) *S. cerevisiae* (PDB 6HU9) and (B) *M. smegmatis* (PDB 6HWH) supercomplexes. In (A), distances for the FeS center in the C (FeS_C_) and B (FeS_B_) positions, respectively (see inset), are indicated in the two halves of the complex III_2_ dimer. Note that the arrangement shown in (A) is a fusion of two different structures where the FeS center is either in FeS_B_ (left monomer) or FeS_C_ (right monomer) (B position PDB is 1KYO, C position is PDB 3H1H). The positions of cyt. *c* bound to cyt. *bc*_1_ or Cyt*c*O are indicated (cyt. *c* at complex III is PDB 1KYO, cyt. *c* at complex IV is PDB 5IY5), see also ref (37). In (B), the open and closed conformations of the cyt. *cc* domain, observed in a single supercomplex, are shown (SodC is PDB 1PZS).

Recent progress in development of methods to isolate pure respiratory supercomplexes has allowed functional studies using biochemical and biophysical techniques, previously employed in studies of the individual respiratory complexes. Major advancement in the field was contributed by the use of cryo-EM to determine the overall architecture of supercomplexes, high-resolution structures of their components as well as positions and distances between all cofactors (shown for the *S. cerevisiae* and *M. smegmatis* supercomplexes in Figure 3). These studies are still in an early phase, but the data available to date allows a discussion of possible links between the molecular architecture and function of respiratory supercomplexes. This review is centered around the *S. cerevisiae* supercomplex, but we also discuss the *M. smegmatis* and *C. glutamicum* obligate III_2_IV_2_ supercomplexes while focusing on functional similarities and differences to the mitochondrial counterpart. The emphasis is put on the biological processes at the molecular level in terms of physical mechanisms.

## 2 Complex III

**Figure 4:**
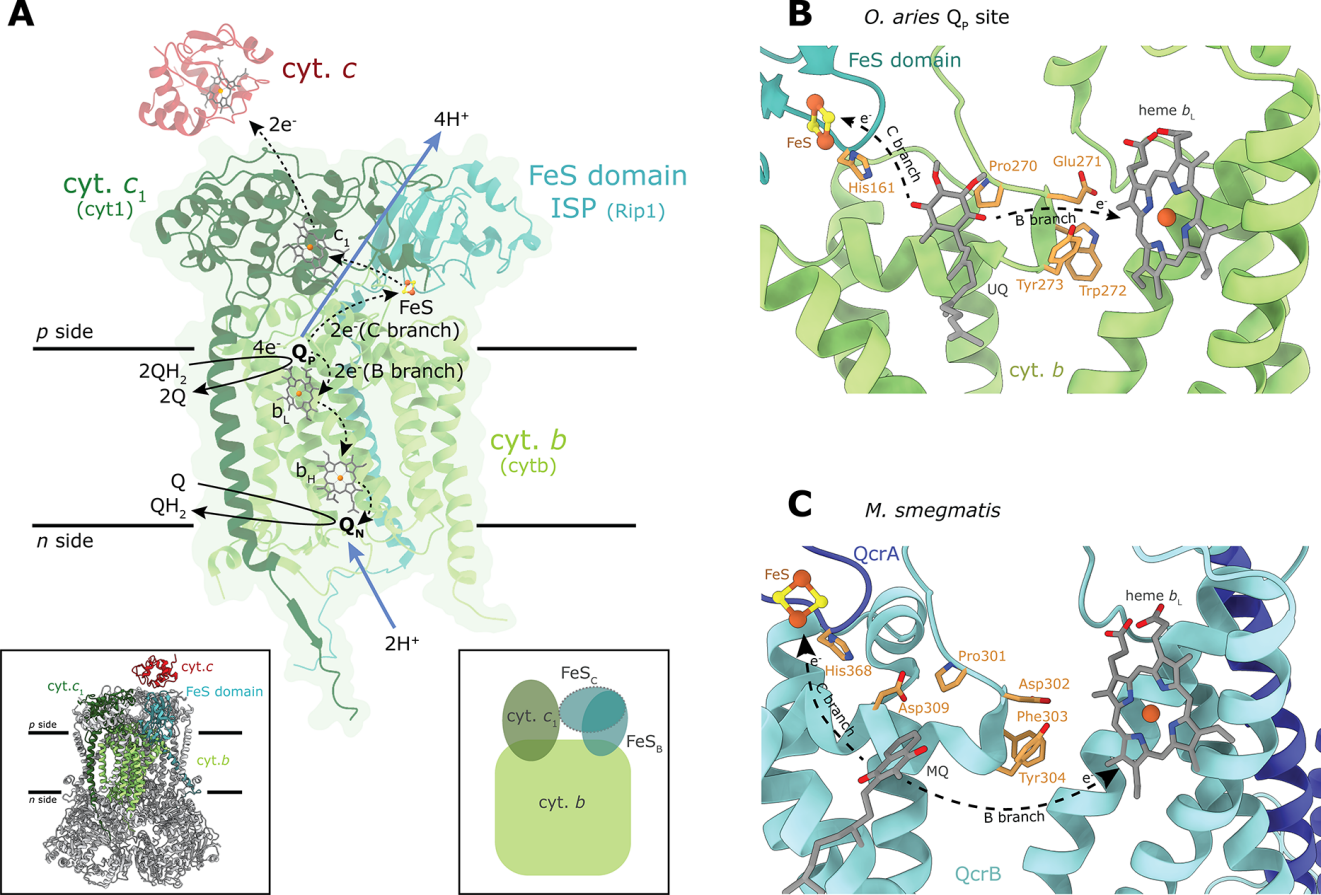
Complex III. (A) The catalytically important subunits of one monomer of complex III_2_ (cyt. *bc*_1_) from *S. cerevisiae* (PDB 6HU9) and the catalyzed reaction. The electron-transfer paths along the B and C branches are indicated with dashed lines, while proton uptake and release are shown with blue arrows. Note that the total stoichiometry of electron and proton transfer is indicated for oxidation of two QH_2_. Upon oxidation of each QH_2_ in the Q_P_ site, two electrons are transferred, one electron along each of the B and C branches, respectively. One H^+^ is transferred to His161 (His181 in *S. cerevisiae*) ligand of the FeS center (shown in B) and is transferred to the *p* side upon movement of the FeS-domain from the B position (FeS_B_ in the right-hand side inset to A) to the C position (FeS_C_). The second H^+^ is transferred via protonatable residues of the cyt. *b* subunit (see text). The same sequence of electron and H^+^ transfer is repeated upon binding of the second QH_2_ in the Q_P_ site. The inset on the lower left shows all subunits of the dimer, including accessory subunits in gray and bound cyt. *c* (PDB 1KYO). In main panel A, the FeS center is found in an intermediate B/C position. (B) The Q_P_ binding site of *O. aries* (sheep, PDB 6Q9E) with a bound ubiquinone (UQ),^38^ the only structure of a mitochondrial cyt. *bc*_1_ in which the Q_P_ site is occupied by Q. The Q_P_ site and all functionally important residues are conserved in the *S. cerevisiae* cyt. *bc*_1_. (C) The Q_P_ site of *M. smegmatis* complex III (PDB 6ADQ).

Complex III (cyt. *bc*_1_) is an obligate homodimer. Each monomer is composed of three main, functionally important catalytic subunits (Figure 4A): (*i*) cyt. *b* (QcrB in actinobacteria), which harbors two hemes B and two quinone-binding sites; (*ii*) cyt. *c*_1_, which harbors a heme C (QcrC, which harbors two hemes C in actinobacteria); (*iii*) the Rieske iron–sulfur protein (ISP, called QcrA in actinobacteria or Rip1 in *S. cerevisiae*), which harbors a 2Fe-2S center (FeS) that is bound in an ectodomain on the *p* side of the membrane (reviewed, e.g., in ref (78−85)). In addition to these three catalytic subunits, in *S. cerevisiae*, each cyt. *bc*_1_ monomer is composed of an additional 7 subunits (Figure 2A), collectively shown in gray in the inset to Figure 4A (lower left).

### 2.1 Catalytic Reaction and Quinone Binding

Complex III catalyzes net oxidation of QH_2_ and reduction of cyt. *c* in a reaction sequence that is referred to as the proton-motive Q-cycle, which contributes to maintaining the proton electrochemical potential across the inner mitochondrial membrane.^86^ The QH_2_ electron donor binds in a Q-binding site referred to as Q_P_, which is located near the *p* side of the membrane (also called Q_o_) (Figure 4A). In the mitochondrial cyt. *bc*_1_, this site is characterized by a conserved PEWY (Pro-Glu-Trp-Tyr) motif^87^ (residues 270–273 in Figure 4B). The equivalent in *M. smegmatis* is PDFY (PDVY in *C. glutamicum*) residues 301–304 in Figure 4C. The first electron from QH_2_ is transferred to the FeS center and then to cyt. *c*_1_ along a branch that is referred to as the “C branch” (Figure 4A). This electron transfer is accompanied by release of two protons to the aqueous solution on the membrane *p* side. The second electron is transferred along the “B branch”, consecutively to the low-potential heme *b*_L_, the high-potential heme *b*_H_ and a Q in the Q_N_ site (also called Q_i_), which forms a semiquinone, SQ^•–^. After oxidation of QH_2_ in the Q_P_ site, the product Q is replaced by another QH_2_, and the sequence of electron and proton-transfer reactions is repeated. As a result, a doubly reduced QH_2_ is formed at the Q_N_ site after proton uptake from the *n* side. The QH_2_ is released from the Q_N_ site by equilibration with the Q/QH_2_ pool in the membrane. The overall reaction catalyzed by cyt. *bc*_1_ is (see also Figure 4A):

Oxidation of first QH_2_ in the Q_P_ site:
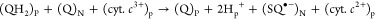
1a

Oxidation of second QH_2_ in the Q_P_ site:
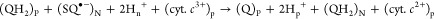
1b

Overall reaction:
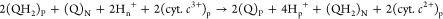
1cwhere subscripts *n* and *p* refer to the two sides of the membrane, respectively, and *N* and *P* refer to the two Q-binding sites, respectively.

Crystal structures of cyt. *bc*_1_ complexes have revealed a single bound Q in the Q_N_ site for each monomer, but the Q_P_ site is typically empty. The putative position of the Q_P_ site was instead revealed by the location of inhibitors such as stigmatellin or myxothiazol (reviewed in refs (78,81,84)). In the cryo-EM structures of the *S. cerevisiae* cyt. *bc*_1_ complexes^33−35,37^ a Q could not be modeled convincingly in the Q_P_ site, but a ubiquinone (UQ) was found to be bound in the Q_N_ site, in line with the earlier structural studies using X-ray crystallography. A recent cryo-EM study of the mammalian I_1_III_2_ supercomplex^88^ revealed a UQ in the Q_P_ site, but only in one monomer of the cyt. *bc*_1_ dimer (the other Q_P_ site was empty). In another recent cryo-EM structure of complex III_2_ from *C. albicans*, density for a UQ was found in both Q_P_ sites of the dimer (as well as in the Q_N_ sites), although at low occupancy.^89^

On the basis of the observation of an empty Q_P_ site and a UQ bound in the Q_N_ site in the *S. cerevisiae* complex III, it was recently suggested that a higher affinity for UQ at the Q_N_ site would prevent release of a semiquinone that would give rise to superoxide upon reaction with O_2_.^35^ However, we note that (*i*) the difference in affinity for UQ at the two binding sites is not directly related to the affinity of the negatively charged semiquinone radical, SQ^•–^, at these sites,^90^ (*ii*) SQ^•–^ is not released to the membrane, i.e., the reaction of O_2_ with SQ^•–^ is more likely to occur *in situ*, but (*iii*) it occurs at the Q_P_ rather than at the Q_N_ site.^81,91,92^ We instead suggest that observation of a bound UQ in the Q_N_ site reflects a higher affinity for the substrate UQ in that site, compared to the product UQ in the Q_P_ site (all structures were obtained with the oxidized state of complex III).

In the *M. smegmatis* and *C. glutamicum* III_2_IV_2_ supercomplexes, menaquinone (MQ) was observed in the Q_P_ and Q_N_ sites but also at additional sites on the *p* side of complex III.^43−45^ The MQ in the Q_P_ site of the *M. smegmatis* complex III overlaps in space with that of UQ in the mammalian complex III. In *C. glutamicum*, the Q_P_ cavity is larger than in *M. smegmatis*, and the data suggest that MQ could also occupy a position just outside of the Q_P_ site, suggesting two possible binding modes, one inside and one just outside of the Q_P_ site.^45^ Furthermore, in both *M. smegmatis*(44) and *C. glutamicum*(45) supercomplexes, clear density corresponding to an additional MQ on the *p* side was observed. In the *M. smegmatis* supercomplex, this MQ is positioned near the Tyr of the PDFY motif, at the vertex of a triangle formed the FeS center (at a distance of ∼20 Å) and heme *b*_L_ (at a distance of ∼20 Å). In the *C. glutamicum* supercomplex structure, the second MQ is located at a distance of ∼14 Å from heme *b*_L_ and ∼35 Å from the FeS center. The role of an additional MQ binding site on the *p* side is unknown, but identification of these Q-binding sites in both *C. glutamicum* and *M. smegmatis* suggests a functional role, for example, to bypass energy conservation in complex III at low O_2_ concentrations.^45^

### 2.2 The Bifurcated Electron Transfer

A bifurcated electron transfer from QH_2_ at the Q_P_ site is required by the Q-cycle mechanism. As outlined above, in this process, one electron from QH_2_ is transferred to FeS and one to heme *b*_L_ along the C and B branches, respectively (Figure 4A), which is schematically outlined in the following equation, assuming a putative semiquinone intermediate:
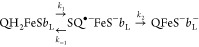
2

#### 2.2.1 Canonical Complex III

The detailed mechanism of this bifurcation at the Q_P_ site remains enigmatic.^78,81,82^ Transfer from QH_2_ to FeS with a midpoint potential *E*_m_^7^ ≥ 300 mV is thermodynamically more favorable than transfer to heme *b*_L_ with *E*_m_^7^ ≅ 0 mV (when heme *b*_H_ is oxidized). Thus, oxidation of QH_2_ results first in reduction of FeS along the C branch. The second electron could in principle also be transferred along the same C branch to FeS after reoxidation of FeS^–^ by cyt. *c*_1_, i.e., without energy conservation.^82^ Instead, the electron is transferred along the B branch in a reaction that is strictly controlled yielding almost complete reduction of heme *b*_L_. This phenomenon was clearly illustrated in an experiment where transfer to the Q_N_ site, along the B branch, was inhibited by binding of the Q_N_-site inhibitor antimycin. Even though, in principle, the enzyme could turnover by electron transfer via the C branch only, this block of the B branch resulted in reduction of both hemes *b*_L_ and *b*_H_ and almost full inhibition of the cyt. *bc*_1_ turnover complex.^93^

Crystal structures of canonical cyt. *bc*_1_ complexes revealed that the FeS ectodomain could adopt different positions where in the two extreme orientations the FeS cluster is found in proximity to either cyt. *c*_1_ (C position) or heme *b*_L_ (B position).^94−96^ These two FeS ectodomain positions are indicated schematically in the right-hand side inset to Figure 4A (see also inset to Figure 3A). The distance spanned by the FeS cluster while moving between the B and C positions is almost 20 Å, and the structural data suggested that the FeS cluster could accept electrons from QH_2_ (in site Q_P_) only in the B position, while electron transfer to cyt. *c*_1_ would occur only in the C position. However, the link between Q/QH_2_ binding in the Q_P_ site, the redox state of FeS and the equilibrium constant for the two FeS-domain positions remains enigmatic.^78,81,97^

Structural studies with different types of inhibitors bound in the Q_P_ site indicate that the position of the FeS ectodomain depends on its interactions with the inhibitor as well as minor structural changes caused by the inhibitor binding.^78,89,95,97−103^ There are two classes of Q_P_-site inhibitors referred to as P_f_ (*f* for fix) and P_m_ (*m* for mobile), respectively. The P_f_ class of inhibitors, such as the UQ analogue stigmatellin, fix the FeS ectodomain in the B position, presumably due to formation of a hydrogen bond between the inhibitor and the FeS ectodomain. The P_m_ class of inhibitors, such as, e.g., myxothiazol or azoxystrobin, displace the FeS ectodomain from the B position yielding a mobile domain that adopts different positions, including the C position. A recent cryo-EM study with the P_m_-type fungal complex III_2_ inhibitor Inz-5 revealed the distribution of these positions.^89^

Crystal structures of complex III_2_ revealed also intermediate positions of the ectodomain, in between the B and C positions.^104^ This variability in the ectodomain position was explained by differences in crystal packing (summarized in ref (84)). However, in the cryo-EM structures of the *S. cerevisiae* cyt. *bc*_1_(34,35) the FeS ectodomain also adopts an intermediate position (shown in Figure 4A), i.e., the intermediate ectodomain position is not a consequence of protein crystallization. Interestingly, in a recent cryo-EM structure of the *C. albicans* cyt. *bc*_1_ several classes of particles were observed in which the FeS head domain is either in the B position, C position, or in between these positions,^89^ suggesting a statistical distribution of these states, which is consistent with spectroscopic data.^292^ Similarly, in the cryo-EM structure of the *R. capsulatus* cyt. *bc*_1_, subpopulations were identified with the FeS ectodomain either in the B or C position with an empty Q_P_ site.^46^ In the cryo-EM structure of the mammalian cyt. *bc*_1_, only one Q_P_ site of complex III_2_ dimer is occupied,^88^ but the FeS domain adopts the C position in both monomers. Furthermore, in the recently determined structure of the plant supercomplex from *Vigna* (*V*.) *radiata*, both FeS domain positions were observed in the absence of bound Q in the Q_P_ site.^32^ Hence, all these data suggest that the position of the FeS ectodomain is stochastic when the Q_P_ site is empty or occupied by an oxidized Q.^78,81,88,97^ On the other hand, binding of a reduced hydroquinone in the Q_P_ site when the FeS cluster is oxidized may shift the equilibrium of the FeS domain toward the B position, similarly to binding of stigmatellin.^89,105−110^

Because movement of the FeS domain is involved in transfer of the first electron from QH_2_ to cyt. *c*_1_, the equilibrium constant and/or time constant for the FeS domain transition between the B and C positions determines the kinetics of this electron transfer.^82,84^ A stochastic FeS domain movement after oxidation of QH_2_ in the Q_P_ site implies that the B–C transition is not required to accomplish the electron bifurcation from the Q_P_ site,^107^ i.e., electron branching in the Q-cycle is possible without movement of the FeS domain. Indeed, the FeS domain is permanently fixed near the B position in the *M. smegmatis* and *C. glutamicum* III_2_IV_2_ supercomplexes.^43−45^ Rich and colleagues^107^ discussed the thermodynamics and kinetics of electron bifurcation in the framework of eq 2 above and concluded that the mechanism could be explained by a concerted two-electron oxidation of QH_2_.

#### 2.2.2 *M. smegmatis* and *C. glutamicum* Supercomplexes

In the *M. smegmatis* supercomplex, the cyt. *cc* domain of complex III displayed two conformations in the two halves of the supercomplex, a closed conformation in which it is located near the electron acceptor at complex IV, and an open conformation where the electronic connection between the two complexes is interrupted^44^ (Figure 3B). We hypothesized that movement of the cyt. *cc* domain, instead of movement of the FeS ectodomain, could mediate electron transfer from MQH_2_ within the supercomplex.^44^ However, at this point, it is unknown whether or not the cyt. *cc* domain movement is stochastic or linked to other reactions. In the *C. glutamicum* supercomplex^45^ as well as in another structure of the *M. smegmatis* supercomplex,^43^ all elements of the electron-transfer chain appear to be fixed, which suggests that the Q-cycle can be realized without any domain movements. Collectively, these data suggest a variability in the structural solution to a mechanistic realization of the Q cycle, which is discussed in the next subsection.

### 2.3 Proton Release from the Q_P_ Site

#### 2.3.1 Canonical Complex III

The electron bifurcation from QH_2_ along the C and B branches, respectively, is functionally linked to proton release to the membrane *p* side.^82,87,97,110−114^ In the canonical cyt. *bc*_1_, binding of QH_2_ at the Q_P_ site has been suggested to shift the equilibrium of the FeS head domain toward the B position where one of the QH_2_ protons would form a hydrogen bond with the FeS ligand His161 (mammalian complex III numbering, His181 in *S. cerevisiae*). It is well established that upon transfer of the first electron from QH_2_ to FeS, the first proton is transferred to this His161.^82,87,97,111−114^ The second proton has been suggested to be transferred to Glu271 (Glu272 in *S. cerevisiae*) of the PEWY motif (Figure 4B), followed by rotation of the protonated Glu271 toward the heme *b*_L_ propionate upon electron transfer to heme *b*_L_ (Figure 4B). After transfer of the second electron along the B branch, the FeS head domain would transiently adopt the C position (see discussion in the previous section), from where the first electron is transferred to cyt. *c*_1_, linked to proton release from His161 to the *p* side of the membrane. In other words, this mechanism implies that part of the proton-transfer route for the first proton would involve the rotation of the FeS head domain.

It is likely that a spatial distribution of the two proton-transfer paths and the link between proton and electron transfer yields the bifurcated proton transfer. While the transfer route of the first proton from QH_2_ is relatively well characterized, the route of the second proton remains to be explored. The proton from Glu271 has been suggested to be transferred consecutively to Arg79 (not shown in Figure 4B) and the *p* side aqueous phase.^111^ However, functional studies of structural variants at position Glu271 indicate that this residue is not a unique proton acceptor from QH_2_,^115,116^ and there are presumably alternative proton-release pathways.^81^ In the structure of *S. cerevisiae* complex III, residues Glu272 and Tyr274 (equivalent of Asp302 and Tyr304, respectively, in *M. smegmatis*, Figure 4BC), together with other residues, coordinate a network of water molecules between heme *b*_L_ and the Q_P_ site, which may be involved in proton transfer, and determines the dielectric environment of the site.

#### 2.3.2 *M. smegmatis* and *C. glutamicum* Supercomplexes

The mechanism described above outlines that deprotonation of His161 to the *p* side occurs only when the FeS head domain had moved to transiently adopt the electron donating C position. Because in *M. smegmatis* and *C. glutamicum* the FeS domain is fixed in the B position, a different proton-release route is presumably utilized in these complexes. In complex III from *M. smegmatis* and *C. glutamicum*, a Q was found to be bound in a site equivalent to the canonical Q_P_ site.^43−45^ His368, the equivalent of His161, is presumably the acceptor of the first proton from QH_2_ also in these complexes III (Figure 4C). In the *M. smegmatis* complex III, the equivalent of Glu271 is a shorter side chain Asp302, which cannot approach the Q_P_ site sufficiently closely to act as an acceptor of the second proton. Instead, Asp309 (*M. smegmatis* numbering) is found in proximity to the second proton of QH_2_ (Figure 4C). Furthermore, Asp309 is found at ∼4 Å from His368, suggesting a possible common proton-release route of the two QH_2_ protons.^45^ Many actinobacteria harbor a Glu residue instead of Asp309, which could also serve as a proton acceptor.

On the basis of this analysis of the structure, we speculated that a possible Q-cycle mechanism in *C. glutamicum* and *M. smegmatis* complex III may involve the following sequence of events:^45^ (*i*) transfer of the first proton/electron to His368/FeS, (*ii*) transfer of the second proton/electron to Asp309/heme *b*_L_, (*iii*) electron transfer from heme *b*_L_ to heme *b*_H_, linked to deprotonation of Asp309, and (*iv*) electron transfer from FeS to the nearest cyt. *c*_I_ of the cyt. *cc* domain.

The electron transfer from FeS to cyt. *c*_I_ in (*iv*) is assumed to occur only if it is linked to deprotonation of the FeS ligand His368, which is possible only after deprotonation of Asp309, i.e., *after* electron transfer from heme *b*_L_ to heme *b*_H_. Indeed, the electron transfer in (*iii*), from FeS to cyt. *cc* along the C branch, was shown to be rate-limiting for turnover of the *C. glutamicum* supercomplex,^65^ i.e., it would occur after electron transfer along the B branch. In addition, on the basis of analysis of one of the *M. smegmatis* supercomplex structures, we hypothesize that the transition between the open and closed conformation of the cyt. *cc* domain (Figure 3B) may provide a mechanism to gate electron transfer from complex III to complex IV.^44^ However, as indicated above, it is presently unclear how this movement would be linked to the binding of QH_2_ at the Q_P_ site and the proton-transfer reactions. It should be stressed that the mechanism outlined above is based on analyses of structures and is presented only to serve as a guide in the design of experiments aimed at testing this hypothesis.

## 3 Complex IV^117^

**Figure 5:**
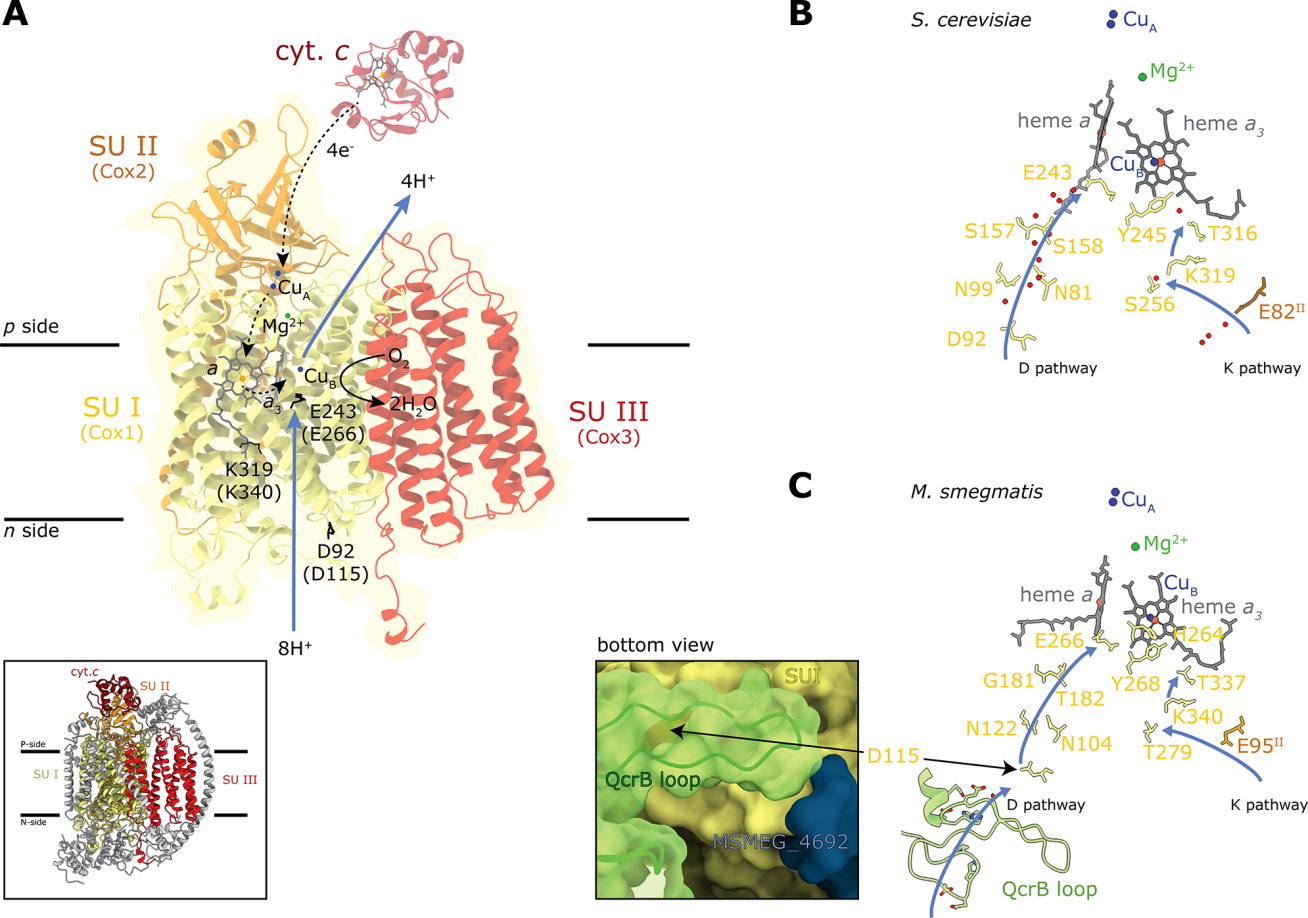
Complex IV. (A) The core subunits of the *S. cerevisiae* Cyt*c*O (complex IV, PDB 6HU9) and the catalyzed reaction. The inset shows all subunits of the *S. cerevisiae* Cyt*c*O, including accessory subunits in gray and bound cyt. *c* (based on the cyt. *c* position in the bovine Cyt*c*O, PDB 5IY5, which displays the same geometry as the *S. cerevisiae* cyt. *c*-Cyt*c*O cocomplex^37^). The D and K proton pathways of the *S. cerevisiae* (B) and *M. smegmatis* (PDB 6HWH) (C) Cyt*c*Os. In (B), water molecules seen in the crystal structures of bacterial and mammalian Cyt*c*Os are included. They were not resolved in the cryo-EM structures of the *S. cerevisiae* Cyt*c*O. (C) The QcrB “lid” of complex III, which covers the D pathway of Cyt*c*O in the *M. smegmatis* supercomplex. Amino acid residue side chains of QcrB that provide an alternative entry pathway to D115 are shown (along the blue arrow below the D pathway).

The mitochondrial complex IV is a member of the heme-copper oxidase family, which is characterized by a catalytic site that is composed of a heme group and a copper ion where dioxygen is reduced to water. Other oxidases, such as the UQH_2_-O_2_ oxidoreductases, cytochrome *bd*(118,119) and alternative oxidases^120^ also catalyze reduction of O_2_ to water in respiratory chains, but these oxidases harbor catalytic sites of different composition and do not belong to the heme-copper oxidase family. The heme-copper oxidase family is defined by homology in subunit I (Figure 5A), which harbors six conserved histidine residues that coordinate three redox-active metal sites: (*i*) a six-coordinated heme group with two axial His ligands (heme *a* in Figure 5A); (*ii*) a five-coordinated heme group with one axial His ligand (heme *a*_3_ in Figure 5A); and (*iii*) a copper ion called Cu_B_, which is coordinated by three His ligands. The latter heme and Cu_B_ form a catalytic site where O_2_ binds and is reduced. In bacteria, the two heme groups may be of the same or different types: hemes *a*, *b*, or *o*. In mitochondria both hemes are of the same *a* type, hence these complexes are sometimes also referred to as cytochromes *aa*_3_.

The heme-copper oxidase family can be divided in two functional subgroups, based on the origin of the electron donor: quinol oxidases and Cyt*c*Os. The former receive electrons from membrane-soluble QH_2_, while the latter receive electrons from cyt. *c*. The quinol oxidase from, e.g., *E. coli* (cytochrome *bo*_3_) has an overall structure that is similar to those of bacterial Cyt*c*Os but lacks the electron acceptor metal site (Cu_A_, see below) and instead harbors a Q-binding site at which QH_2_ donates electrons.

The primary electron acceptor of the mitochondrial Cyt*c*Os, including that of *S. cerevisiae*, is a dinuclear Cu-center called Cu_A_, located near the *p* side in subunit II (Figure 5A). Because electrons from cyt. *c* are donated at the *p* side of the membrane, while protons are taken up from the opposite, *n*, side of the membrane, the reaction yields a charge separation across the membrane that is equivalent to moving one positive charge from the *n* to the *p* side. In addition, for each electron transferred to the catalytic site, one proton is pumped from the *n* to the *p* side, thereby increasing the total charge-separation stoichiometry. The proton-pumping stoichiometry varies between Cyt*c*Os from different organisms. Thus, the reaction catalyzed by the Cyt*c*Os is
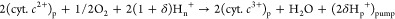
3where δ is the proton-pumping stoichiometry, i.e., number of H^+^ pumped per electron transferred to O_2_, typically 0.5 ≤ δ ≤ 1 (δ = 1 for mitochondrial Cyt*c*Os), subscripts *n* and *p* refer to the two sides of the membrane, and the subscript “pump” refers to pumped protons released on the *p* side (for more detailed reviews on the structure and function of Cyt*c*Os, see refs (121−130)).

It is worth noting that supercomplexes composed of cyt. *bc*_1_ and Cyt*c*O catalyze the same reaction as that catalyzed by quinol oxidases mentioned above, i.e., oxidation of QH_2_ and reduction of dioxygen to water. However, the energy-conservation efficiency is larger for the supercomplex than for, e.g., the *E. coli* cyt. *bo*_3_ because, in addition to the charge separation and proton pumping by the Cyt*c*O part, in the supercomplex there is also a transmembrane charge separation generated by cyt. *bc*_1_.

### 3.1 The Core Subunits

Bacterial heme–copper oxidases consist typically of two to four subunits. The minimum functional unit is composed of subunits I and II, which harbor all four redox-active cofactors that catalyze the reaction in eq 3. Subunits I–III (Cox1–3 in *S. cerevisiae*, Figure 5A) are often referred to as the “catalytic core” because upon removal of subunit III, many Cyt*c*Os lose their activity during turnover, referred to as suicide inactivation (reviewed in ref (131)). The subunit I–III catalytic core is conserved and structurally almost identical in Cyt*c*Os from mammals, yeast, and many aerobic bacteria.

On the basis of an analysis of amino acid sequence homology as well as functionally important structural features, e.g., proton pathways (see below and ref (132)), the Cyt*c*Os have been classified into three major families named A, B, and C.^133,134^ Type A includes the mitochondrial as well as the “mitochondrial-like” bacterial cytochromes *aa*_3_, e.g., from *P. denitrificans*, *R. sphaeroides*, and *M. smegmatis*. Type B includes e.g. the *Thermus* (*T*.) *thermophilusba*_3_ Cyt*c*O, while type C includes the *cbb*_3_ oxidases found, e.g., in *R. sphaeroides*, *R. capsulatus*, and *P. denitrificans*, where a subunit with a diheme cyt. *c* is the primary electron acceptor instead of Cu_A_.^132^

The A family Cyt*c*Os have two well characterized proton-transfer pathways; the K-pathway named after a conserved Lys (K319 or K340, *S. cerevisiae* or *M. smegmatis* numbering, respectively, Figure 5B,C), and the D-pathway named after a conserved Asp at its entrance (D92 or D115 in Figure 5B,C). The A-family is further divided into two subfamilies, A1 and A2. The former is characterized by a subunit I motif “XGHPEVY”, found in, e.g., the mitochondrial Cyt*c*Os including that from *S. cerevisiae*, where “E” is Glu243 in the D proton pathway (Figure 5B), “H” is a ligand of Cu_B_ (His241, not shown in Figure 5B), while “Y” is a catalytically active Tyr245 in the catalytic site. The imidazole group of His241 and the phenol group of Tyr245 (Y) are linked by a covalent bond. Similarly, the *M. smegmatis* Cyt*c*O belongs to the A1 subclass. Subclass A2 instead harbors an “YSHPXVY” motif where the Glu is replaced by a Tyr-Ser pair (“YS”) at about the same position in space in the D pathway.

Subunit I in the *S. cerevisiae* (Cox1) Cyt*c*O comprises 12 transmembrane (TM) α-helices. Subunit Cox2 is composed of two TM α-helices and a head domain, which harbors the redox-active Cu_A_ site (Figure 5A). Subunit Cox 3 is composed of seven TM α-helices that form a V-shaped cleft, which has been suggested to funnel O_2_ from the membrane to the catalytic site.^129,135^ The putative O_2_ channel in Cox 3 typically harbors three tightly bound lipid molecules, PG, PC, and PE, resolved in crystal structures of Cyt*c*O from *R. sphaeroides*, *P. denitrificans*, and *B. taurus*.^136^ In the *S. cerevisiae* Cyt*c*O, two lipid molecules could be modeled in this cleft.^33,34^

### 3.2 The Catalytic Reaction and Proton Pathways

**Figure 6:**
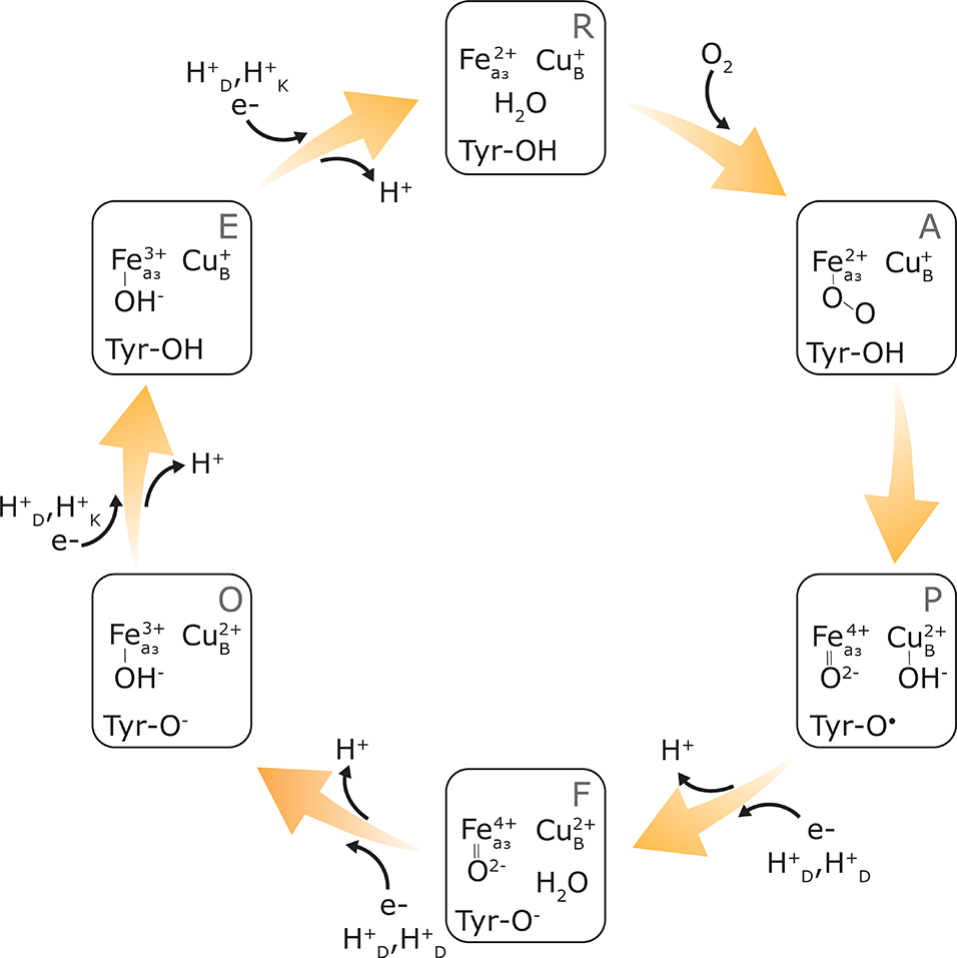
Reduction of O_2_ at the catalytic site of Cyt*c*O. The first electron (e^–^) from cyt. *c* to the oxidized Cyt*c*O (state **O**) is transferred to Cu_B_ to form state **E**. It is accompanied by proton uptake from the *n* side solution though the K pathway (H_K_^+^) to Tyr245 (*S. cerevisiae* Cyt*c*O numbering, Tyr in the figure). Transfer of the second electron to heme *a*_3_ and a proton through the K pathway to a hydroxide at heme *a*_3_ leads to formation of state **R**, where the catalytic site is reduced by two electrons. Next, O_2_ binds to heme *a*_3_ forming state **A**. After transfer of one electron and one proton from the Tyr residue, a ferryl state is formed, called **P** (“peroxy”, for historical reasons). Transfer of the third electron is accompanied by proton uptake through the D pathway (H_D_^+^) and formation of the ferryl state, **F**. After transfer of the fourth electron and another proton through the D pathway to the catalytic site, the oxidized state **O** is formed again. The four transitions **P** → **F**, **F** → **O**, **O** → **E**, and **E** → **R** are each associated with pumping of one proton across the membrane. These protons are taken up through the D pathway (H_D_^+^, each proton released to the *p* side is indicated as H^+^).

During turnover of Cyt*c*O, electron transfer from cyt. *c* to the Cu_A_ site is followed in time by electron transfer to heme *a* and the heme *a*_3_-Cu_B_ catalytic site. Figure 6 illustrates schematically the reaction cycle of the mitochondrial Cyt*c*Os. The oxidized state of Cyt*c*O is referred to as state **O**. Electron transfer from reduced cyt. *c* to the oxidized Cyt*c*O results in reduction of first Cu_B_ and heme *a*_3_, which is associated with uptake of two protons from the membrane *n* side through the K proton pathway (see Figure 5B) to the catalytic site. Each electron transfer from cyt. *c* to the catalytic site is associated with proton pumping across the membrane. The two-electron reduced catalytic site binds O_2_ (state **A**), which results in breaking the O–O-bond by electron transfer from heme *a*_3_ and Cu_B_ as well as hydrogen transfer from Tyr245, which forms a radical (state **P**). In the following reaction steps one electron is transferred to the catalytic site in each of the **P** → **F** and **F** → **O** transitions. Each of these reduction steps is linked to uptake of two protons from the *n* side through the D proton pathway, one to the catalytic site and one is pumped across the membrane. The branching point from which the substrate and pumped protons are transferred along different trajectories is located at Glu243.

The structure and function of the K and D proton pathways have been studied in detail in bacterial A1-type Cyt*c*Os,^129,130,137−150^ and their involvement in proton uptake also confirmed for the *S. cerevisiae* mitochondrial Cyt*c*O.^151,152^ The K pathway starts near Glu82 in subunit II at the membrane *n* side (Figure 5B). It is connected via a water molecule to Ser256, which is hydrogen-bonded to the conserved Lys319. Proton transfer from the Lys residue requires a conformational change of the side chain toward the catalytic site.^135^ From the “up-position” the proton is transferred, via a water molecule and Thr316 to Tyr245 at the catalytic site (see Figure 5B).

Residue Asp92 of the D pathway is positioned at the inside of a cleft at the *n*-side surface of subunit I. The pathway is composed of polar residues that coordinate ∼10 H_2_O molecules, which span the distance of ∼20 Å from Asp92 to Glu243 (Figure 5B). The maximum rate of proton uptake to the catalytic site, via the D pathway, is ∼10^4^ s^–1^ at pH 7, and it drops with increasing pH displaying a p*K*_a_ of 9.4,^153^ which is attributed to titration of Glu243^153^ (but, see ref (154)). Replacement of the Asp or Glu residues by their nonprotonatable analogues, Asn or Gln, respectively, result in impaired activity and a complete block of proton uptake.^137,142,155−159^

Minor structural changes around the orifice of the D pathway influence the proton-uptake kinetics and proton pumping stoichiometry. For example, one-residue changes at Asp92 or in the vicinity of this residue in bacterial and *S. cerevisiae* Cyt*c*Os result in lower proton-pumping stoichiometry or complete uncoupling of proton pumping from the O_2_-reduction reaction, often without altering the Cyt*c*O turnover or proton-uptake rate.^151,153,160^ Similarly, changes in the surface-exposed loop of subunit I in the *R. sphaeroides* Cyt*c*O, outside of Asp92, yielded modified pH dependence and uncoupling of proton pumping.^161^ Also, removal of *R. sphaeroides* subunit III, which has a loop of residues near Asp92 (*S. cerevisiae* numbering), resulted in a dramatic shift in the pH dependence of the proton-uptake rate^162^ and allowed proton uptake via alternative surface protonatable groups, other than Asp92^163^ (the two subunit I and III loops are found just below D92/D115 in Figure 5B,C, but are not shown in the figure). Collectively, these data indicate that moderate alteration of the D pathway near the entry point modulate proton-pumping stoichiometry and result in changes in the pH dependence of the proton-transfer kinetics through the D pathway.^164^

Interestingly, in the *M. smegmatis* and *C. glutamicum* III_2_IV_2_ supercomplexes, in addition to the subunit III (subunits CtaE/F) loop, another loop that extends from cytochrome *b* (QcrB subunit of complex III) covers the orifice of the D pathway^44^ and presents an alternative route for proton entry into the D pathway, via protonatable groups of the QcrB loop^45^ (Figure 5C, the subunit III loop is not shown in the figure, it is positioned between Asp115 and the QcrB loop). As outlined above, the D pathway entrance is highly conserved and the proton-uptake kinetics is controlled by an intricate web of interactions between the pathway residues. A modified architecture as a result from supramolecular interactions between complexes III and IV in the *C. glutamicum* and *M. smegmatis* III_2_IV_2_ supercomplexes suggests that proton uptake by complex IV could be modulated by structural changes in complex III.^65^

In the mammalian Cyt*c*O, a third proton pathway (H pathway) was suggested based on a structural analysis.^121,139^ In bacterial Cyt*c*Os, the equivalent of this pathway is not involved in proton transfer.^165^ Structural analyses and data from functional studies of structural variants in which putative residues of the H pathway were modified in the mitochondrial *S. cerevisiae* Cyt*c*O do not support a functional role of this pathway.^151,152,166^ Furthermore, key residues of the suggested H pathway are not present in Cyt*c*O from plant mitochondria,^32^ which suggest that its involvement in proton pumping would have to be restricted to the mammalian Cyt*c*Os.

### 3.3 Peripheral subunits of the *S. cerevisiae* Cyt*c*O

In addition to the three core subunits Cox1–3, the *S. cerevisiae* Cyt*c*O is also composed of nine peripheral subunits called Cox4–9, Cox12, Cox13, and Cox26,^33,34^ where the latter was identified only recently^167,168^ (Figure 2A). All of these accessory subunits, except Cox26, have subunit homologues in mammals. Some of these subunits have been suggested to be involved in regulation of the electron transfer and proton pumping activities of the Cyt*c*O.^169−172^ A discussion of the role of all these subunits is beyond the scope of this review, but we briefly discuss those accessory subunits that are relevant in the context of supramolecular interactions with cyt. *bc*_1_. A detailed description of all the accessory subunits in *S. cerevisiae* Cyt*c*O is found in ref (173) (see also ref (171)).

Subunit Cox5 is the major interaction partner with cyt. *bc*_1_ in the *S. cerevisiae* supercomplex (Figure 2A). It is homologous to mammalian CoxIV and is expressed as one of two isoforms, called Cox5A or Cox5B, which share 68% sequence identity.^173^ Expression of the two isoforms depends on the oxygen concentration; the former version is expressed at normoxic conditions (∼200 μM O_2_), while the latter is expressed at low oxygen concentrations (<0.5 μM).^174,175^ Early data indicated that the catalytic turnover of Cyt*c*O is higher with Cox5B than with Cox5A.^176^ However, more recent data indicate that the elevated Cyt*c*O activity is not simply a result of replacement of Cox5A by Cox5B because a genetic replacement of Cox5A by Cox5B did not yield any differences in the turnover activity nor of the affinity for O_2_ or cyt. *c*.^177^

In the *S. cerevisiae* Cyt*c*O, subunit Cox13 is composed mainly of a single bow-shaped TM α-helix at the periphery of Cyt*c*O.^34^ In the cryo-EM structural model, it interacts with Cox1, Cox3, and Cox12 on the *p* side and with Cox4 on the *n* side of the membrane^34^ (Figure 2A).

### 3.4 The *M. smegmatis* Cyt*c*O

The *M. smegmatis* Cyt*c*O core is composed of subunits CtaD (subunit I), CtaC (subunit II), as well as CtaE and CtaF, which together form the equivalent of subunit III. The structure of this subunit I–III core is very similar to that of the canonical Cyt*c*O. In addition, the *M. smegmatis* supercomplex harbors a number of accessory subunits (Figure 2B).^43,44^ Even though some of these subunits are attached only to the Cyt*c*O part of the supercomplex, we consider them being components of the supercomplex rather than of Cyt*c*O itself. Furthermore, as already mentioned above, in the *M. smegmatis* supercomplex subunit QcrB of complex III is extended to interact with complex IV. Figures 2B and 3B show the open and closed positions of the cyt. *cc* domain (QcrC) in the two halves of the supercomplex.

### 3.5 Nonredox Active Metal Sites

In addition to the redox-active metal sites, A-type Cyt*c*Os harbor a number of nonredox active metal sites (Figure 5A). An Mn^2+^/Mg^2+^ (depending on the concentration of the metal in the growth medium) is located near the catalytic site of mammalian and bacterial A-type Cyt*c*Os^139,178,179^ and was also identified in one cryo-EM structure of the *S. cerevisiae* Cyt*c*O.^34^ In addition, a Ca^2+^/Na^+^ site was confirmed in the *S. cerevisiae* Cyt*c*O^34^ (see also refs (139,179,180)). These metal sites are presumably also present in the actinobacterial supercomplexes.^45^ Furthermore, a Zn^2+^ ion is bound in Cox4 of the *S. cerevisiae* Cyt*c*O^34^ (see also ref (139)). Added Zn^2+^ also binds near the proton pathways to slow or impair proton uptake.^181−185^

### 3.6 The Putative Cyt*c*O Dimer

**Figure 7:**
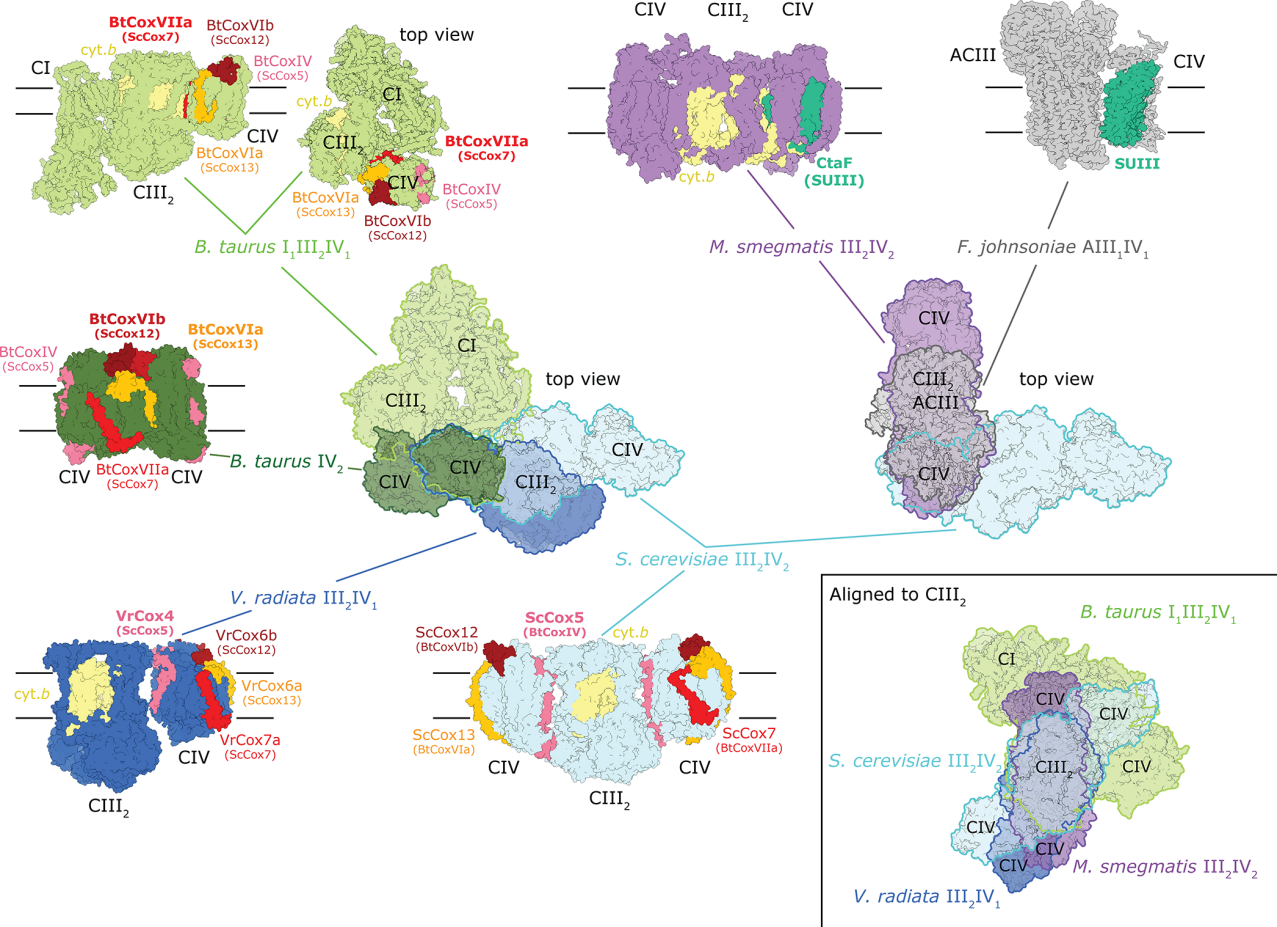
Arrangement of supercomplexes with known structures that contain complexes III and IV in different species. The *B. taurus* (cow) Cyt*c*O dimer is also shown (PDB 1OCC), other references are given in Table 1. The main panel in the middle shows the alignment of complex III_2_ relative to the position of complex IV with its subunits at the interface of complex III_2_ indicated in bold text and in different colors. All interacting subunits for all supercomplexes are marked in specific colors for reference in order to indicate their relative positions in all supercomplexes. The prefixes Bt (*B. taurus*), Sc (*S. cerevisiae*), and Vr (*V. radiata*) are added because of the different subunit numbering used for the equivalent subunits in different organisms. The mitochondrial supercomplexes are shown on the left, while the obligate *M. smegmatis* III_2_IV_2_ and the alternative complex III–IV supercomplexes are shown on the right. The *S. cerevisiae* supercomplex is encircled by a blue line; it is shown as reference for both the mitochondrial and bacterial supercomplexes. Top views and side views with approximate positions of the membrane with black lines. The inset shows the same supercomplexes but aligned to the complex III_2_ dimer (alternative complex III in *F. johnsoniae* is not shown here).

The bacterial Cyt*c*Os are typically monomers. The first crystal structures of the mammalian Cyt*c*O revealed a dimer,^139^ which is consistent with earlier data from functional studies suggesting that formation of the dimer would be functionally relevant.^171^ As seen in Figure 7, in the mammalian Cyt*c*O, the equivalent of subunit Cox12 and Cox13 in *S. cerevisiae*, i.e., subunits CoxVIb and CoxVIa, are found at the monomer–monomer interface in the crystal structure of the dimeric enzyme (interface subunits are marked in bold text in Figure 7). Here, the CoxVIa subunit adopts a structure different from that of Cox13 in *S. cerevisiae*.^139,186^

More recent structural and functional studies showed that the O_2_-reduction activity of the Cyt*c*O monomer was not significantly different from that of the dimer, and only minor structural differences were observed between the monomeric and dimeric forms.^187^ Furthermore, recent structures of supercomplexes composed of complexes I, III, and IV (sometimes also referred to as respirasomes) from mammals showed that the Cyt*c*O bound in these preparations is a monomer^38−41^ (Figure 7), as also seen for supercomplexes *in situ* in mammals, yeast, and plants.^29^

In *S. cerevisiae*, almost all Cyt*c*O is found in supercomplexes.^17,72^ In variants with only one Cyt*c*O (III_2_IV), the enzyme is obviously a monomer, but also in the III_2_IV_2_ variant, the two Cyt*c*Os are maximally separated in the supercomplex (Figure 2A). The current data also suggest that the small fraction free Cyt*c*O in *S. cerevisiae* mitochondria is found in monomeric form.^17,72,188^ Even though a fraction of Cyt*c*O dimer was observed upon reconstitution of the *S. cerevisiae* Cyt*c*O in liposomes,^189^ this observation may be consequence of detergent solubilization of the enzyme prior to reconstitution in a membrane as well as a lipid composition that differs from that of the inner mitochondrial membrane.

## 4 Complex III–IV Supercomplexes

### 4.1 The *S. cerevisiae* Supercomplex

The interface surface between cyt. *bc*_1_ and Cyt*c*O within the *S. cerevisiae* supercomplex is surprisingly small,^24^ with a main part of the cyt. *bc*_1_–Cyt*c*O interactions on the matrix (*n*) side of the supercomplex where the N-terminal domain of Cox5 binds to Cor1^34^ (Figure 2). In addition, the C-terminal domain of Cox5 on the *p* side of the membrane interacts with the C terminus of Qcr6 and with the cyt. *c*_1_ domain (Figure 2). The first supercomplex structures^34,35^ were determined with the Cox5A isoform (in ref (34), Cox5B was removed genetically). Many of the residues of Cox5A that are involved in binding to cyt. *bc*_1_ in the supercomplex are the same in the two isoforms of Cox5. Accordingly, a recent structural study of supercomplexes composed of Cyt*c*O with either Cox5A or Cox5B did not show any isoform-dependent interactions.^33^

Only minor structural changes result from formation of the supercomplex. The data suggest that the N terminus of the TM α-helix of the Rieske iron–sulfur protein (Rip1) in cyt. *bc*_1_ undergoes a conformational change upon interactions with a cardiolipin molecule within the supercomplex.^34^ However, the authors also noted that this change would not impact the FeS-containing head domain of the iron–sulfur protein,^34^ i.e., the function of cyt. *bc*_1_ is unlikely to be altered as a result of supercomplex formation. Furthermore, the structural comparison of the N terminus of the iron–sulfur protein was made to the crystal structure of cyt. *bc*_1_, i.e., any differences in interactions with cardiolipin may also reflect differences in the organization of cyt. *bc*_1_ in crystals and in the cryo-EM sample, respectively. The conformation of the other cyt. *bc*_1_ subunits that interact with Cyt*c*O (mainly Cor1, but also cyt. *c*_1_ and Qcr8, see Figure 2A) are not altered by the supramolecular interactions.^33,34^ Another difference in structure possibly caused by the supramolecular interaction is the configuration of the N-terminal domain of Cox5A. This protein segment may bind ATP, which has been suggested to allosterically regulate the Cyt*c*O activity.^190^ Because upon forming a supercomplex this domain is shifted toward cyt. *bc*_1_, the structural difference may be a consequence of binding of Cyt*c*O to cyt. *bc*_1_ within the supercomplex.^34,35^ However, because a structure of the *S. cerevisiae* Cyt*c*O alone (i.e., not part of a supercomplex) is not available, the structural comparison of subunit Cox5A was made for the equivalent subunit of the isolated mammalian (bovine heart) Cyt*c*O and the *S. cerevisiae* Cyt*c*O in a supercomplex.^34^ Therefore, the structural difference may reflect that of the equivalent subunits in the different Cyt*c*Os. We also note that the turnover activity of free Cyt*c*O is the same as that of Cyt*c*O in a supercomplex with cyt. *bc*_1_,^37^ which suggests that the putative structural changes seen upon supercomplex formation are not functionally relevant. In conclusion, because the supramolecular interaction surface is small and any structural differences that may occur upon supercomplex formation are minor,^33−35^ the activities of cyt. *bc*_1_ and Cyt*c*O are unlikely to be “regulated” upon formation of the supercomplex.

As indicated above, the monomer–monomer interface in the mammalian dimer^139^ involves subunits CoxVIa and CoxVIb^139^ (Figure 7). The equivalent subunits in the *S. cerevisiae* Cyt*c*O, Cox13, and Cox12, respectively, were suggested to define a monomer–monomer interface also in a putative dimer of the *S. cerevisiae* Cyt*c*O.^173^ Because in the *S. cerevisiae* supercomplex the cyt. *bc*_1_–Cyt*c*O interface involves subunit Cox5, subunits Cox12 and Cox13 are exposed on the opposite side of the Cyt*c*O (see Figures 2 and 7). Therefore, if a Cyt*c*O dimer would be formed in *S. cerevisiae* by interactions through Cox12 and Cox13, a chain of supercomplexes would form in the membrane. Indeed, such a multisupercomplex structure was suggested by Schägger for yeast and mammalian mitochondria.^18^ However, to our knowledge, there is no published data in support of such a scenario. Furthermore, Hartley *et al*. noted that the bow-shaped topology of Cox13 would hinder dimerization of Cyt*c*O.^34^ In addition, the suggested binding of the respiratory supercomplex factor 2 (Rcf2, see below) at Cox13 would probably also prevent Cyt*c*O dimerization through interactions via Cox13.^33^

### 4.2 Other (I)III_2_IV_1/2_ Supercomplexes

Figure 7 shows known structures of supercomplexes in which complexes III and IV are in direct contact (see also Table 1), as well as the mammalian complex IV dimer. The orientation of the mitochondrial respiratory complexes in relation to complex IV is shown in the main left-hand side panel, with Cyt*c*O subunits that interact with the other complexes indicated in different colors (bold text is used to indicate interactions for each supercomplex). To the right are shown bacterial complex III-IV supercomplexes with known structures. The inset on lower right shows an overlay of all supercomplexes but instead aligned to the complex III_2_ dimer.

As seen in Figure 7, there is a great variability in the relative orientation of complexes III_2_ and IV, i.e., the interaction surfaces of these complexes in supercomplexes varies between different organisms. In the mammalian I_1_III_2_IV_1_ supercomplex,^38^ the surface of the homologous subunits of complex III that interact with complex IV in the *S. cerevisiae* supercomplex, instead bind to complex I. In this mammalian supercomplex, main interactions with cyt. *bc*_1_ occur via Cyt*c*O subunit CoxVIIa (Cox7 in *S. cerevisiae*). The details of the cyt. *bc*_1_–Cyt*c*O interactions in *S. cerevisiae* as well as interactions within the mammalian Cyt*c*O dimer are discussed in the previous sections.

In the plant supercomplex from *V. radiata* mitochondria the approximate relative orientation of complexes III_2_ and IV is similar to that of *S. cerevisiae*. However, the protein–protein interaction sites differ and the orientation angle differs by 18° (defined by heme *b*_H_s in complex III_2_, and hemes *a* and *a*_3_ in complex IV, Figure 7).^32^ As with the *S. cerevisiae* supercomplex, subunit Cox5 (Cox4 in *V. radiata* mitochondria) faces toward complex III. However, on the matrix side the interactions between Cox5 and Cor1, observed in *S. cerevisiae*, are absent in *V. radiata* because the equivalent of Cox5 in the latter is shorter by ∼100 amino acid residues at the N terminus. Instead, the main interactions are found on the cytosolic side between *V. radiata* Cox4 and Qcr6, which are more extensive in the *V. radiata* than in the *S. cerevisiae* mitochondrial supercomplex.^32^

In the *M. smegmatis* III_2_IV_2_ supercomplex the main III_2_–IV interactions are mediated via complex IV subunits CtaE and CtaF, which together form the equivalent of Cyt*c*O subunit III, and QcrB (cytochrome *b*) of complex III_2_, which is also bound to complex IV via the extended QcrB loop on the periplasmic (*n*) side (Figure 7).^43,44^

In the structure of the *F. johnsoniae* supercomplex composed of an alternative complex III and Cyt*c*O, interactions are mediated via the Cyt*c*O subunit III.^47^ The authors noted that this subunit III lacks TM α-helices 1 and 2, i.e., consists of five TM α-helices. These five TM α-helices are equivalent to subunit CtaE of the *M. smegmatis* Cyt*c*O, which also interact with complex III_2_ in this supercomplex. As noted above, in *M. smegmatis*, the equivalents of TM α-helices 1 and 2 are present and formed by the CtaF subunit. This observation shows that subunit III of Cyt*c*O displays a structural variability that may be adopted to accommodate different interaction partners.^47^

The variability in the interaction surfaces of complexes III and IV most likely excludes a universal structure–function modulation that would be a consequence of III_2_–IV supercomplex formation in mitochondria. The situation is different for actinobacterial supercomplexes where formation of the III_2_IV_2_ supercomplex introduces new architecture to otherwise conserved structural elements, for example, those involved in proton uptake and pumping in complex IV.

### 4.3 Cardiolipin in Supercomplexes

Cardiolipin is typically found in membranes that are involved in energy conversion, i.e., that maintain an electrochemical proton gradient.^191−193^ The phospholipid is unique in having a dimeric structure consisting of two phosphatidyl moieties linked to glycerol and four acyl chains. The p*K*_a_ values of the two phosphate groups were reported to be different with one p*K*_a_ being above 8.0, i.e., the cardiolipin headgroup would carry only one negative charge at neutral pH.^194^ The high-p*K*_a_ headgroup was suggested to act as a proton trap near enzymes that maintain or utilize electrochemical proton gradients.^194^ However, results from more recent studies indicate that the two p*K*_a_s are similar (≤ ∼3) and that cardiolipin carries two negative charges at neutral pH.^195,196^

In mammalian cells, cardiolipin is found primarily in the mitochondrial inner membrane where the weight fraction of the lipid is ∼18%^193^ (16% in the *S. cerevisiae* inner mitochondrial membrane^197^). In addition, the lipid may be enriched in the inner leaflet of the inner mitochondrial membrane,^191^ and it has been suggested to be involved in shaping the cristae.^52^ Cardiolipin has been identified as an integral part of many membrane proteins,^198,199^ and the enzymatic activities of, for example, detergent-solubilized mitochondrial cyt. *bc*_1_ and Cyt*c*O are dependent on the presence of bound cardiolipin^200,201^ (this effect is not observed with the *R. sphaeroides* Cyt*c*O^202^). In addition, cardiolipin is involved in apoptosis, where one step in the cascade of signaling reactions involves formation of a co-complex between the lipid and cyt. *c*, which results in cyt. *c* acquiring peroxidase activity.^203^

**Figure 8:**
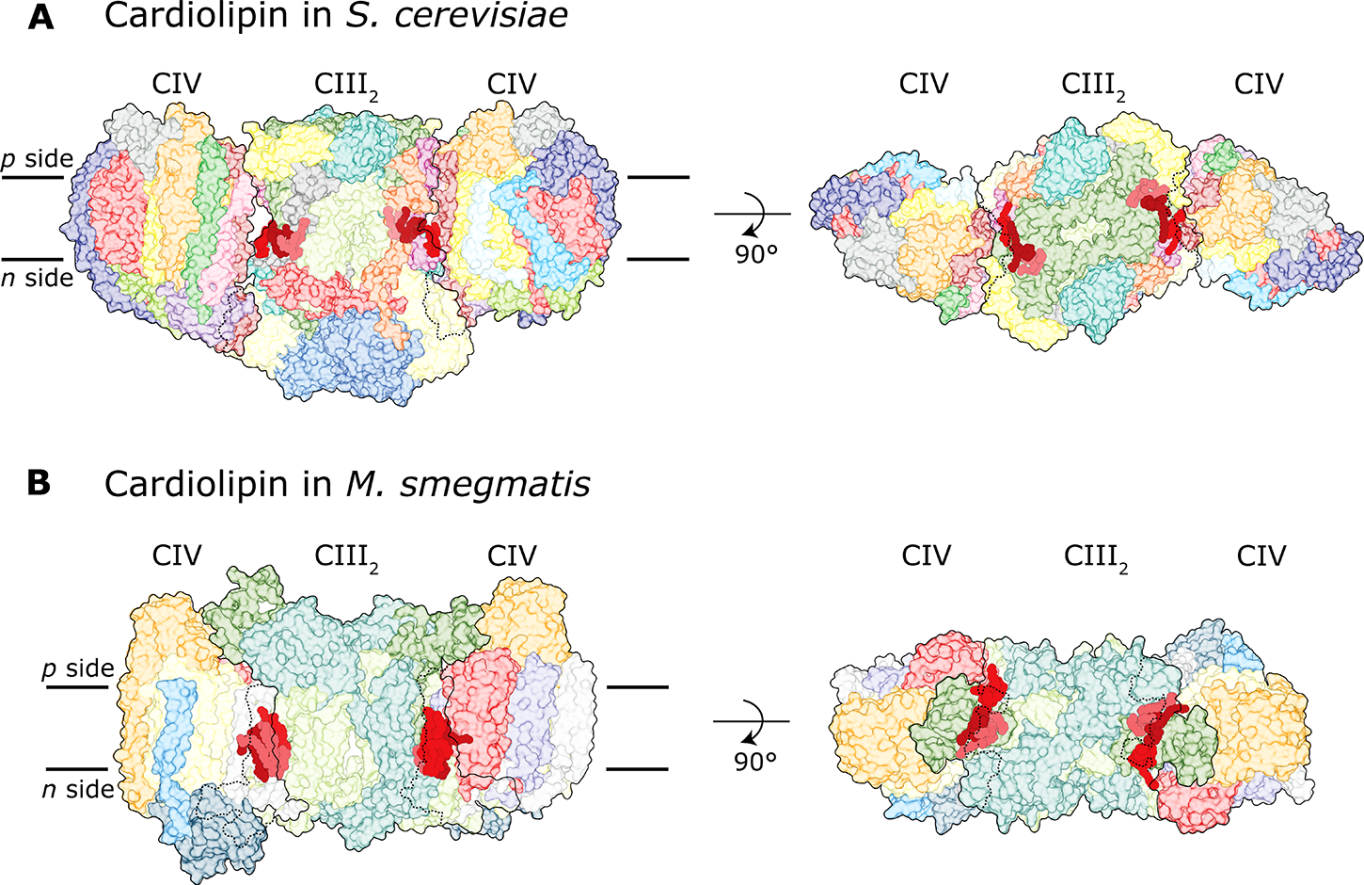
Cardiolipin in complex III–IV supercomplexes. All cardiolipin (shown in red) head groups face the *n* side. The boundaries of complexes III and IV are indicated by solid lines. The dashed lines indicate boundaries on the opposite side of each supercomplex. (A) The *S. cerevisiae* supercomplex. Subunits are colored as in Figure 2A. (B) The *M. smegmatis* supercomplex. Subunits are colored as in Figure 2B.

A discussion on the role of cardiolipin in supporting enzymatic activities of the respiratory complexes and its involvement in apoptosis is beyond the scope of this review. Instead, we discuss briefly cardiolipin’s role in maintaining supramolecular interactions between cyt. *bc*_1_ and Cyt*c*O in supercomplexes. The lipid is enriched in both the mammalian I_1_III_2_IV_1_^204^ and *S. cerevisiae* III_2_IV_1/2_^205^ supercomplexes. In the presence of cardiolipin the fraction of supercomplexes is larger than in its absence.^71,204−208^ Recent cryo-EM structures of the *S. cerevisiae* III_2_IV_1/2_ supercomplexes showed that a cardiolipin and presumably a phosphocholine are found at the cyt. *bc*_1_–Cyt*c*O interface. Two other cardiolipins are found in the vicinity where they also may contribute to supporting the cyt. *bc*_1_–Cyt*c*O interaction^34^ (Figure 8A). The lipid is suggested to mediate interactions between cyt. *bc*_1_ and Cyt*c*O acting as a “glue”^209^ by simultaneously binding to specific sites at each of these two complexes.^199,210^

Involvement of cardiolipin in stabilizing binding of cyt. *bc*_1_ to Cyt*c*O may, at least in part, explain why the fraction of supercomplexes and free complexes depends on *S. cerevisiae* growth conditions,^17^ which often influence the lipid composition of mitochondria. Furthermore, it is likely that the fraction of the two supercomplex forms, i.e., III_2_IV_1_ and III_2_IV_2_, is not only determined by the concentration of the cyt. *bc*_1_ and Cyt*c*O components in the membrane,^33^ but also by the presence of cardiolipin,^189,205,206,208,209^ which would modulate the cyt. *bc*_1_–Cyt*c*O binding affinity.

In the obligate III_2_IV_2_ supercomplexes in *M. smegmatis* and *C. glutamicum* three cardiolipins are found at the interface of complexes III and IV (Figure 8B).^44,45^ Similarly, to the *S. cerevisiae* supercomplex, the head groups of all these cardiolipin molecules face the *n* side of the membrane.

### 4.4 Respiratory Supercomplex Factors

Respiratory supercomplex factors, Rcf1 and Rcf2, physically associate with cyt. *bc*_1_ and Cyt*c*O. Both Rcf1 and Rcf2 contain a hypoxia-induced gene domain 1 (HIGD1), which is conserved in a wide range of organisms.^211−214^ In Rcf1, the HIGD1 is in the N terminus and the C terminus has a fungi-specific domain, composed of approximately 60 amino acid residues. In Rcf2, which is a fungi-specific protein, the HIGD1 is located at the C terminus, preceded by a subdomain composed of ∼100 amino acid residues, which forms two transmembrane helices.^215,216^ The Rcf2 protein has been shown to be proteolytically processed to yield a stable C-terminal fragment that associates with Cyt*c*O.^217^

Data from early studies of the functional role of Rcf1 and Rcf2 were interpreted to indicate that these factors are required for formation of the cyt. *bc*_1_–Cyt*c*O supercomplexes in *S. cerevisiae*.^73,188,214,217−220^ The conclusion is in part based on observations that the ratio between supercomplexes and free components decreased upon genetic removal of Rcf1, which was also interpreted to suggest that this factor acts as a bridge between the components of the supercomplex. However, Rcf1 interacts with the Cox3 subunit and possibly also Cox13,^214,219,221−223^ but the recently determined supercomplex structures show that these subunits are found at the opposite side of Cyt*c*O from the III_2_–IV interaction surface (Figure 2A).^33−35,37^ Hence, Rcf1 cannot bridge supramolecular interactions between cyt. *bc*_1_ and Cyt*c*O. Similarly, a recently determined cryo-EM structure suggested binding of Rcf2 at the distal side of the supercomplex.^33^

More recent studies suggest that Rcf1 is instead involved in assembly of Cyt*c*O (reviewed in refs (54,224)) and incompletely assembled Cyt*c*O would result in a smaller fraction of supercomplexes. In other words, the cyt. *bc*_1_–Cyt*c*O supercomplexes can form also in the absence of Rcf1, but when Rcf1 is removed, a fraction of Cyt*c*O is modified, which yields less supercomplexes. Similarly, the Aim24 protein in *S. cerevisiae*(225) and mammalian homologue of Rcf1, HIGD2A, have recently been shown to be involved in the assembly of Cyt*c*O.^226,227^ It is interesting to note that data from recent studies indicate that removal of Rcf1 or Rcf2 affects the ability of the Cyt*c*O to maintain a proton electrochemical potential across the membrane, possibly due to proton leaks across the incorrectly assembled fraction of Cyt*c*O in the absence of Rcf.^228^

Genetic deletion of Rcf1 yields a subpopulation of Cyt*c*O that is incorrectly assembled and a subpopulation that is correctly assembled.^219,229−231^ In the absence of Rcf1, the correctly assembled Cyt*c*O subpopulation displays a lower activity and a modified heme *a*_3_-Cu_B_ catalytic site.^229−231^ The activity of this subpopulation could be restored upon addition of recombinantly expressed Rcf1,^232^ which suggests that in the correctly assembled Cyt*c*O reversible binding of Rcf1 can modulate the Cyt*c*O activity. This finding is further supported by recent data showing that Rcf1 positively modulates Cyt*c*O activity also in the intact mitochondrial membrane.^221^

Deletion of Rcf2 alone has a small effect on Cyt*c*O turnover,^214,219,221,233,234^ but more recent data indicate that binding of Rcf2 results in lowering the Cyt*c*O activity.^221^ Collectively, these data suggest that, in addition to being involved in assembly of Cyt*c*O, the binding of the Rcf proteins is linked to changes in the turnover activity.

Mass spectrometry revealed the presence of Rcf1 and Rcf2 in preparations of purified *S. cerevisiae* supercomplexes, but these proteins were not resolved in the first cryo-EM structures.^34,35^ As indicated above, more recent cryo-EM data show additional density in a pocket formed by Cox1, Cox3, Cox12, and Cox13 that in the supercomplex containing the Cox5B isoform could be assigned to the processed C terminus (HIGD1) of Rcf2.^33^ In Cyt*c*O containing the Cox5A isoform, the additional density could not be assigned with confidence. As the HIGD1 fragment is conserved to both Rcf1 and Rcf2, but is found in the C terminus of Rcf2 or the N terminus of Rcf1, the interaction between this segment and a putative conserved CytcO site would expose the remaining parts of the two Rcf proteins to different sides of HIGD1 (discussed in more detail in ref (215)). In other words, any additional interactions with the supercomplex would be very different for the Rcf1 and Rcf2 proteins. This observation reveals how binding of Rcf1 and Rcf2 could differently modulate the activity of CytcO or the supercomplex. An interaction between the homologous bovine HIGD1A protein and bovine CytcO was also observed.^235^ Furthermore, formation of the mammalian III_2_IV supercomplexes is dependent on another protein factor, COX7A2L.^27,28^

It is also interesting to note that interaction of Rcf1 with subunit Cox3 (subunit III) may modulate O_2_ binding at catalytic site^221,228,234^ because Cox3 harbors the lipid-containing V-shaped cleft suggested to be used for O_2_ diffusion from the membrane phase into the Cyt*c*O catalytic site. Data from earlier studies with the *R. sphaeroides* Cyt*c*O showed that changes in lipid molecules in this cleft result in changes of the Cyt*c*O catalytic site.^236^

As evident from the discussion above, the Rcf proteins determine the structure and function of complex IV of the *S. cerevisiae* respiratory chain, however, their role at the molecular level is complex and presently not fully understood.

### 4.5 Superoxide Dismutase in the *M. smegmatis* Supercomplex

A copper-containing superoxide dismutase (SodC) dimer subunit was found to be bound in the *M. smegmatis* III_2_IV_2_ supercomplex, near the cyt. *cc* head domain of the QcrC subunit^43,44^ (Figure 2B). As other SOD enzymes, it catalyzes the dismutation of the O_2_^•–^ radical to H_2_O_2_ and O_2_:
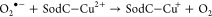
4a
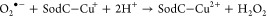
4b

The functional role of this SodC is unknown. Because the semiquinone formed as an intermediate at the Q_P_ site of complex III may react with O_2_ to form superoxide,^81,91,92^ association of a SodC with the respiratory supercomplex could allow detoxification near the O_2_^•–^ generation site.^44^ In addition, the product H_2_O_2_ released by the SodC is a substrate for Cyt*c*O, which upon transfer of two electrons from cyt. *c* reduces H_2_O_2_ to water.^237^ Alternatively, the reduced Cu^+^ formed in SodC in the first reaction step (eq 4a) may transfer an electron to cyt. *cc* and then to Cu_A_ in Cyt*c*O, where it would enter the respiratory chain thereby bypassing formation of H_2_O_2_.^44^ In some anaerobic organisms, an essentially opposite reaction is catalyzed by a superoxide reductase, which reduces O_2_^•–^ to H_2_O_2_ upon electron transfer from an external donor.^238^ Recently, an integral-membrane superoxide oxidase was discovered in *E. coli*.^239^ The *M. smegmatis* SodC has a similar orthologue in *M. tuberculosis*, where the subunit could remove O_2_^•–^ generated by the host as a defense mechanism in the phagolysosomes of macrophages.^44^

## 5 Interaction of Complexes III_2_ and IV with Cytochrome *c*

In mitochondria cyt *c* is a small, typically ∼12 kDa, water-soluble protein that diffuses in the three-dimensional (3D) intermembrane space (Figure 1B). Cytochrome *c* has a dipole moment and a net positive charge.^240,241^ The edge of the heme group is positioned toward the positively charged protein surface, which docks either to cyt. *c*_1_ or near Cu_A_ at negatively charged surfaces of cyt. *bc*_1_ or Cyt*c*O, respectively.^242−244^ The orientation of cyt. *c* is the same when binding to either cyt. *bc*_1_ or Cyt*c*O.^245,246^

It is generally assumed that the intracellular ionic strength is relatively high (80–150 mM), and it has been shown that at this ionic strength a major fraction of cyt *c* diffuses in three dimensions.^16,51^ However, a recent analysis revealed that only the cation concentration is kept at high concentration, while the concentration of small anions is much lower and the remaining negative charges are found at the surfaces of polyanionic macromolecules.^247^ As a consequence, the Debye screening radius in the intracellular medium is larger than that obtained for a monovalent salt electrolyte at 80–150 mM. Oliveberg, Wennerström, and coauthors estimated that a more reasonable mimic of the intracellular environment is the equivalent of ∼20 mM of a 1:1-electrolyte. As a consequence, the electrostatic interactions between the positively charged cyt. *c*, and its negatively charged interaction partners are likely to be much stronger than those observed when mimicking the intracellular environment in a solution containing 80–150 mM monovalent salt. Below, we discuss the consequence of supercomplex-cyt. *c* interactions for electron transfer between complexes III and IV in supercomplexes, but first we briefly describe data from studies of interactions of cyt. *c* with complexes III_2_ and IV, respectively.

### 5.1 Cyt. *c* Binding to Complexes III and IV

Early data from steady-state turnover measurements with the mammalian cyt. *bc*_1_ suggested that cyt. *c* binds at a single site near cyt. *c*_1_.^246^ More recent data from NMR studies of the plant complex III identified an additional low-affinity distal binding site.^248^ In the crystal structure of the *S. cerevisiae* cyt. *bc*_1_–cyt. *c* co-complex, cyt. *c* was found bound to cyt. *c*_1_.^243,249^ In the structure of the *S. cerevisiae* III_2_IV_1/2_ supercomplex–cyt. *c* co-complex (see inset to Figure 4A), the position of cyt. *c* at cyt. *bc*_1_ was only slightly shifted compared to that observed in the crystal structure.^37^

Interactions of cyt. *c* with Cyt*c*O are more complex. Results from studies of the steady-state turnover rate of mammalian Cyt*c*O were interpreted to indicate two cyt. *c* binding sites in Cyt*c*O.^245,250,251^ This observation does not automatically imply the presence of two independent binding sites from which an electron is transferred to Cu_A_. The same data could also be explained in terms of “nonproductive” binding of cyt. *c* that interferes with the “productive” binding site.^252^ However, results from other experiments suggested that two cyt. *c* molecules can simultaneously bind to a monomer of the mammalian Cyt*c*O, with *K*_D_ values of ∼10 nM and ∼1 μM, respectively.^245,250,251^ Furthermore, covalent cross-linking of a cyt. *c* at the high-affinity site only had a minor effect on binding of a second cyt. *c* at the low-affinity site.^253^ Binding at each site presumably results in electron transfer from cyt. *c* to Cu_A_, but electron transfer from cyt. *c* at the high-affinity site is slower than that from the low-affinity site.^253^

Studies of the steady-state activity of the *S. cerevisiae* Cyt*c*O were initially interpreted to suggest binding of two cyt. *c* molecules with equal affinities, *K*_M_ ≅ 100 nM.^254^ However, more recent data revealed an additional *K*_M_ of ∼30 μM,^177^ indicating a similar mechanism of cyt. *c* binding to the mammalian and *S. cerevisiae* Cyt*c*O.

The cryo-EM structure of the III_2_IV_1/2_ supercomplex-cyt. *c* co-complex in *S. cerevisiae*(37) showed that the cyt. *c* binding is similar to that seen in the crystal structure of the equivalent co-complex with the bovine Cyt*c*O^244^ (see inset to Figure 5A).

### 5.2 The Electronic Link between Complexes III and IV

#### 5.2.1 Diffusion in 3D

It is clear that association of cyt. *bc*_1_ and Cyt*c*O to form a supercomplex leads to a decrease in the intercomplex distance. The distance between the electron donor site at cyt. *bc*_1_ and the acceptor site near Cu_A_ at Cyt*c*O within the *S. cerevisiae* supercomplex is ∼60 Å (Figure 3A)^34,35^ (see also refs (72,74)), i.e., too long to yield a catalytically relevant electron-transfer rate through docking of a single cyt. *c* between the electron donor and acceptor sites.^255^ Thus, the question arises whether or not a shorter diffusion distance via the water phase of the intermembrane space (defined as 3D diffusion) would result in a higher QH_2_:O_2_ oxidoreductase activity.^36,50^ Considering a reasonable average distance between independently diffusing cyt. *bc*_1_ and Cyt*c*O in the membrane (∼50 nm, see Figure 1B), the 3D diffusion time of cyt. *c* between these complexes is in the order of 10 μs.^50^ Hence, diffusion of cyt. *c* cannot be rate limiting for electron transfer from QH_2_ to O_2_ because the maximum turnover (*k*_cat_) of cyt. *bc*_1_ and Cyt*c*O in *S. cerevisiae* is ∼10^2^ s^–1^ and ∼10^3^ s^–1^, respectively.^17^ Furthermore, the overall electron flux through the respiratory chain *in vivo* is lower than the lowest *k*_cat_ value of the involved components, in the range 40 s^–1^ to 140 s^–1^ (Michel Rigoulet, personal communication). Nevertheless, the QH_2_:O_2_ oxidoreductase activity is dependent on the concentration of externally added cyt. *c* to mitoplasts^36^ or purified supercomplexes at a cyt. *c*:supercomplex ratio similar to that found *in vivo*,^37^ suggesting that the cyt. *c*-mediated electron transfer is rate limiting.

Results from a recent theoretical study showed that the electron flux between cyt. *bc*_1_ and Cyt*c*O, mediated by 3D diffusion of cyt. *c*, is determined by the equilibration time of cyt. *c* with the cyt. *c* pool in the intermembrane space, rather than by the cyt. *c* diffusion time constant itself.^50^ Furthermore, the data showed that this equilibration time increases with decreasing cyt. *c* concentration, i.e., the lower the cyt. *c* concentration, the stronger the distance dependence on activity. For freely diffusing components, a cyt. *c*:supercomplex ratio of 2–3 and an average cyt. *bc*_1_–Cyt*c*O distance of 50 nm (Figure 1B), this scenario yields a cyt. *c*-mediated QH_2_:O_2_ oxidoreductase activity that is slower than the turnover of cyt. *bc*_1_ and is dependent on the average cyt. *bc*_1_–Cyt*c*O distance. Interestingly, on the basis of the data in ref (256), Maldonado *et al*. estimated that in plant mitochondria the cyt. *c*:supercomplex ratio is one,^32^ suggesting an even stronger cyt. *bc*_1_–Cyt*c*O distance dependence on the QH_2_:O_2_ oxidoreductase activity than in *S. cerevisiae* mitochondria. Taking into consideration the recent finding that the salt concentration equivalent of the intracellular environment is estimated to be ∼20 mM^247^ rather than the 150 mM used in the theoretical study,^50^ the diffusion coefficient for cyt. *c* in mitochondria would be a factor of ∼10^2^ lower^51^ than that used in the theoretical study in ref (50). This effect further emphasizes the kinetic advantage in forming supercomplexes, under the assumption that electron transfer occurs via 3D diffusion.

#### 5.2.2 Diffusion in 2D

Many Gram-negative bacteria, e.g., *R. capsulatus*, *R. sphaeroides*, and *P. denitrificans* harbor a membrane-anchored cyt. *c*_y_ in addition to a water-soluble cyt. *c*.^46,257,258^ A cyt. *c*_y_ homologue is the only cyt. *c* present in *Rickettsia prowazekii*.^257,259^ Restriction of cyt. *c* diffusion to the two-dimensional (2D) space of the membrane surface yields shorter diffusion times than for 3D diffusion at the same concentrations of the involved components.^50^ Furthermore, integration of a membrane-anchored cyt. *c* into a cyt. *bc*_1_–Cyt*c*O supercomplex allows direct electron transfer from the donor at cyt. *bc*_1_ to the acceptor at Cyt*c*O,^59,260^ even though the linker between the membrane domain and the cytochrome domain in cyt. *c*_y_ is too long to distinguish between 2D and restricted 3D diffusion. In a recent study, the normally water-soluble cyt. *c* was attached to a membrane-bound protein in *S. cerevisiae* mitochondria, which allowed electron transfer between complexes III and IV over a time scale similar to that *in vivo*.^261^

Some Gram-positive bacteria, which lack an outer membrane, harbor membrane-associated cyt *c*s that are attached either via a transmembrane polypeptide or a lipid anchor.^262^ In *Bacillus* PS3, a supercomplex composed of cyt. *bc*_1_, Cyt*c*O and a cyt. *c* was identified and shown to display quinol oxidase activity, i.e., electron transfer from quinol to oxygen.^68^ In the Gram-positive actinobacteria from, e.g., *M. smegmatis* and *C. glutamicum* electron transfer between cyt. *bcc* and Cyt*c*O occurs via the diheme cyt. *c* ectodomain of the QcrC subunit of the cyt. *bcc* complex (Figures 2B and 3B). Because these bacteria lack any water-soluble or membrane-anchored free cyt. *c*, a supercomplex composed of cyt. *bcc* and Cyt*c*O is required for electron transfer from MQH_2_ to dioxygen.^62,64,65,263^ Disruption of the supercomplex using detergent results in a decrease in activity.^263^

**Figure 9:**
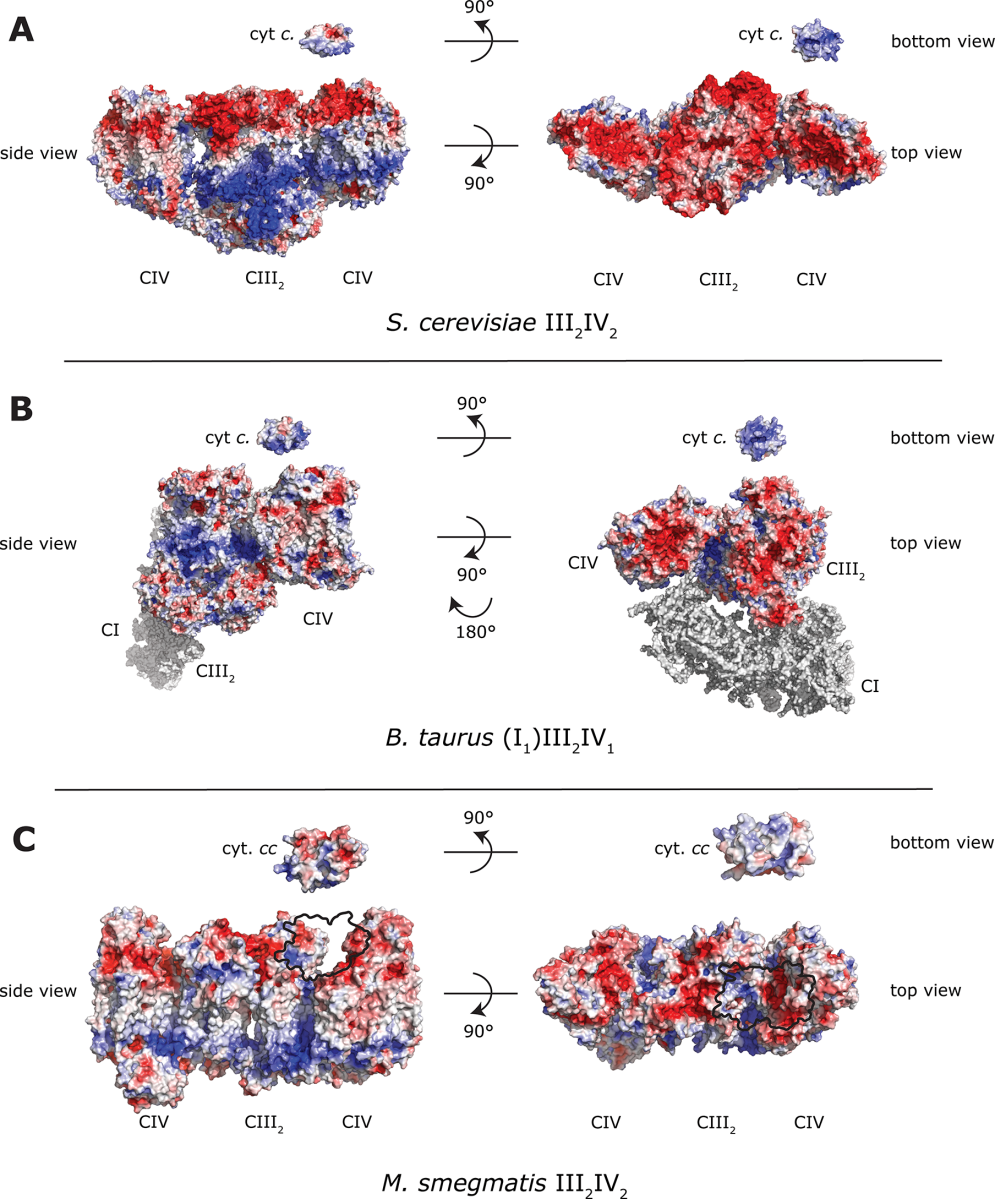
Surface representation of the electrostatic potential in III–IV supercomplexes. The *S. cerevisiae* (PDB 6HU9) (A), *B. taurus* (cow) (PDB 5LUF) (B), and *M. smegmatis* (PDB 6HWH) (C) supercomplexes are shown. Cyt. *c* is from either *S. cerevisiae* (A, PBD 1YCC) or *B. taurus* (B, PDB 2B4Z). For *M. smegmatis* (C), the cyt. *cc* head domain of QcrC in the closed conformation was separated from the supercomplex and the electrostatic potentials were calculated separately for the supercomplex and cyt. *cc* domain, respectively. The original position of the cyt. *cc* domain at the top of the supercomplex is encircled by a black line in (C). Color range from red to blue for an electrostatic potential from −5 to +5 *k*_B_*T*/*q*, where *k*_B_ is the Boltzmann constant, *T* is the absolute temperature, and *q* is a the unit charge. The figure was prepared using the APBS tool^270^ with standard settings of the PyMOL software (*Molecular Graphics System*, version 2.4; Schrödinger, LLC).^271^

Electron transfer between cyt. *bc*_1_ and Cyt*c*O by 2D diffusion of cyt. *c* that is bound to the supercomplex surface or weakly associated with the membrane has been discussed also in organisms that harbor a water-soluble cyt. *c*(37,50,77,264−268) (see also ref (53)). The surface between the cyt. *c*-binding sites at cyt. *bc*_1_ and Cyt*c*O in the *S. cerevisiae* supercomplex is negatively charged (Figure 9A), and one cyt. *c* per Cyt*c*O is tightly bound to the supercomplex^204,234,269^*in situ* (but not in purified complexes). Assuming the same scenario in plant mitochondria, an estimated cyt. *c*:supercomplex ratio of one in *V. radiata*(32) suggests that the entire cyt. *c* pool would be associated with supercomplexes but presumably at equilibrium. Recent Cryo-EM structures of the supercomplex with added cyt. *c* revealed distinct states where cyt. *c* is bound either to cyt. *bc*_1_ or Cyt*c*O, or resides at intermediate positions at the supercomplex surface.^37^ Measurement of the supercomplex activity as a function of the concentration of added cyt. *c* yielded apparent *K*_M_ values of ≤6 nM and ∼1.7 μM, i.e., much smaller than those obtained with isolated *S. cerevisiae* Cyt*c*O (∼100 nM and ∼30 μM, respectively, see above). These data suggest a stronger binding to the supercomplex than to Cyt*c*O, which is consistent with the large negatively charged binding surface for cyt. *c* between cyt. *bc*_1_ and Cyt*c*O. The QH_2_:O_2_ oxidoreductase activity of the supercomplex is ∼20 e^–^/s for a supercomplex with a single bound cyt. *c*. This rate decreased upon dissociation of the supercomplex, i.e., when increasing the average distance between cyt. *bc*_1_ and Cyt*c*O. Collectively, the structural and kinetic data showed that electron transfer within the supercomplex is mediated by 2D diffusion of a single surface-associated cyt. *c*. It is also interesting to note that the rate of electron transfer between cyt. *bc*_1_ and Cyt*c*O with a single bound cyt. *c* is near the lower limit of the electron flux through the respiratory chain *in vivo*. It is also worth mentioning that the above-described experiments were performed at the assumed near-physiological monovalent salt concentration of ∼150 mM, which was also required to prevent protein aggregation on the cryo-EM grids.^37^ Considering the novel finding that a better mimic of physiological conditions is 20 mM monovalent salt,^247^ the cyt. *c*–supercomplex interactions are most likely even stronger *in vivo* than those experimentally observed.^37^

In conclusion, the combined cryo-EM and kinetic data show that supercomplex formation in *S. cerevisiae* does not result in increasing the electron transfer rate by decreasing the cyt. *c* 3D diffusion distance, as recently suggested.^36^ Rather, formation of III_2_IV_1/2_ supercomplexes in *S. cerevisiae* results in switching to a different mechanism that involves 2D diffusion from the electron donor to the electron acceptor.^37^ In other systems electron transfer between complexes III and IV may occur by 3D diffusion and the theoretical studies show that also under these conditions, there is a kinetic advantage in decreasing the intercomplex distance by formation of supercomplexes.^50^ The 2D-diffusion mechanism in *S. cerevisiae* is similar to that suggested for electron transfer from cyt. *bc*_1_ to the *cbb*_3_ Cyt*c*O via a movable membrane-anchored cyt. *c*_y_ domain in *R. capsulatus*.^46^

**Figure 10:**
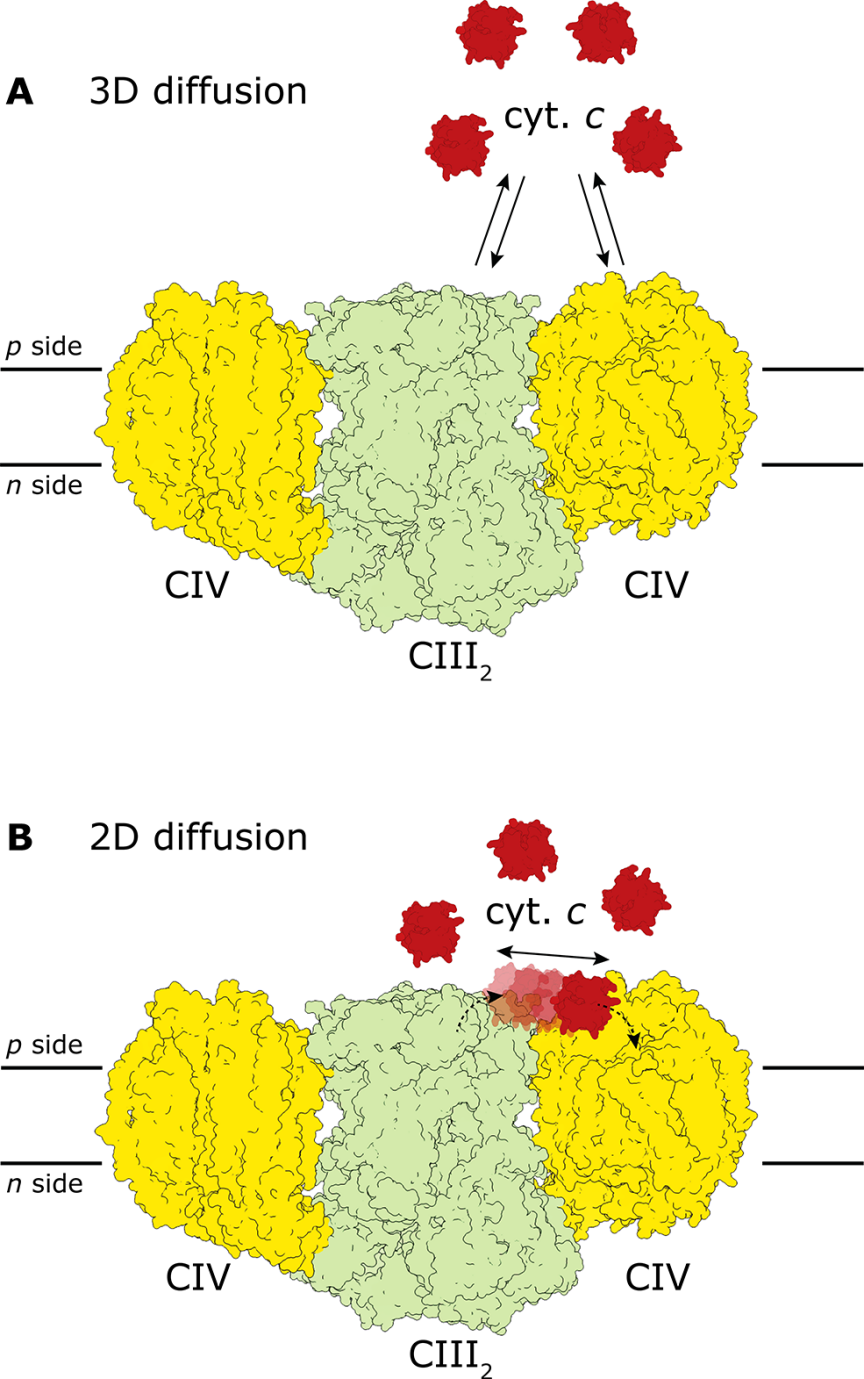
Model for electron transfer from cyt.*bc*_1_ to Cyt*c*O in the *S. cerevisiae* supercomplex. (A) Electron transfer via 3D diffusion of cyt. *c*. (B) Electron transfer via 2D diffusion of cyt. *c*. Note that the surface-attached cyt. *c* is assumed to be in equilibrium with the cyt. *c* pool, but the time constant for equilibration of the surface-attached cyt. *c* with the pool cyt. *c* (as well as electron transfer between the surface-attached cyt. *c* and pool cyt. *c*) is assumed to be slower than diffusion between the binding sites at cyt. *bc*_1_ and Cyt*c*O (modeled after ref (37)). *S. cerevisiae* supercomplex and cyt. *c* are PDBs 6HU9 and 1YCC, respectively.

Electron transfer from cyt. *bc*_1_ to Cyt*c*O by 2D diffusion of cyt. *c* along the supercomplex surface resembles a “substrate channeling” model, which has been criticized based on the finding that cyt. *c* diffusion in *S. cerevisiae* is unrestricted.^269^ However, 2D diffusion of cyt. *c* is not in conflict with this finding because it assumes only weak electrostatic interactions between cyt. *c* and the supercomplex surface, and cyt. *c* remains in equilibrium with the cyt. *c* pool during the electron-transfer process^16^ (see Figure 10).

In mammalian mitochondria, complexes III_2_ and IV are not only part of respirasomes but also assemble independently to form III_2_IV supercomplexes.^27,28,272^ The structure of these supercomplexes is presently not known. Figure 9B shows the electrostatic potential surface of the cyt. *bc*_1_–Cyt*c*O part of the mammalian respirasome. As seen in the figure, the negatively charged cyt. *c* binding sites at cyt. *bc*_1_ and Cyt*c*O are less connected by negative charges on the surface in between the sites than in the *S. cerevisiae* supercomplex (Figure 9A). This difference in charge distribution may reflect the much lower fraction of Cyt*c*O that is part of supercomplexes in mammalian (15–30%^54^) than in *S. cerevisiae* yeast (∼90%,^72^) mitochondria. In other words, in the mammalian respiratory chain electron transfer between cyt. *bc*_1_ and Cyt*c*O occurs primarily via 3D diffusion.

It is also interesting to note that in the *M. smegmatis* III_2_IV_2_ supercomplex, interactions between the movable cyt. *cc* domain^44^ (see Figure 2B) and complex IV most likely occur by electrostatic interactions between positive charges on the cyt. *cc* surface and negative charges at complex IV (Figure 9C). However, the extracellular surface of complex III is positively charged, which indicates that the cyt. *cc* domain is held in place by its TM α-helix rather than by electrostatic interactions.

#### 5.2.3 Effects of Cox5/cyt. *c* Isoforms

Because subunit Cox5 is located at the interface of cyt. *bc*_1_ and Cyt*c*O in the supercomplex^33−35,37^ (Figure 2A), it is positioned at the diffusion path of cyt. *c*. Expression of the two interchangeable isoforms of Cox5, i.e., Cox5A and Cox5B, correlates with the expression of the two cyt. *c* isoforms, iso-1 and iso-2, respectively; Cox5A and iso-1 cyt. *c* are expressed under normoxia, while Cox5B and iso-2 cyt. *c* are expressed under hypoxia.^174,273,274^ This correlation may be coincidental, but we discuss briefly its possible consequences. The supercomplex structure was essentially the same with either Cox5A or Cox5B,^33^ and no effects were observed on the supercomplex activity. In addition, the maximum catalytic activity of Cyt*c*O and its affinity for both cyt. *c* isoforms and O_2_ were unaffected upon replacement of Cox5A by Cox5B.^177^ However, the supercomplex activity was measured at a cyt. *c*:supercomplex ratio of >10^3^,^34^ where the electron-transfer rate saturates at a maximum value, *k*_cat_. It is possible that at the much smaller cyt. *c*:supercomplex ratio of ∼2–3, found in *S. cerevisiae* mitochondria *in vivo* (cf. ref (37)), an effect on the intercomplex electron transfer would be observed depending on cyt. *c* and Cox5 isoforms. In other words, it cannot be excluded that electron transfer between cyt. *bc*_1_ and Cyt*c*O within the supercomplex is regulated by altering the pairwise expression levels of Cox5 and cyt. *c* isoforms.

#### 5.2.4 Binding of cyt. *c* to Rcf1

Cytochrome *c* has also been shown to bind to Rcf1.^232,234,275,276^ The original suggestion that Rcf1 could be found at the interface of complexes III and IV prompted us to suggest that formation of a putative Rcf1–cyt. *c* co-complex would play a similar role to that of cyt. *c*_y_, i.e., mediate electron transfer via a membrane-associated cyt. *c*.^234^ However, this particular consequence of the Rcf1–cyt. *c* interaction appears less likely in *S. cerevisiae* in view of the putative binding of Rcf1 to Cox3/Cox13 (see above), and the position of these subunits at the distal edge of the supercomplex, rather than between cyt. *bc*_1_ and Cyt*c*O (Figure 2A). On the other hand, assuming that Rcf1 would bind at the same position as Rcf2,^33^ cyt. *c* binding to an Rcf1–Cyt*c*O cocomplex would position the cyt. *c* near the cyt. *c*-binding cleft defined by Cyt*c*O subunits Cox12 and Cox2. Interaction of cyt. *c* with Rcf1 at this position would result in increasing the affinity for cyt. *c* to Cyt*c*O to allow electron transfer between complexes III and IV via two transiently bound cyt. *c*s, as discussed previously.^37,264^ Similarly, interaction of cyt. *c* with HIGD1 in mammalian mitochondria has also been observed and discussed.^227,235,277^

As outlined above, the Rcf proteins appear to support a range of functions in respiration, one of which involves binding of cyt. *c*. However, additional data is needed to fully understand the functional significance of the cyt. *c*–Rcf1 interactions at the molecular level.

## 6 Why Supercomplexes?

When considering complexes III and IV, the answer to the question above is rather trivial in the case of the Gram-positive actinobacteria, which do not harbor any water-soluble cyt. *c*. We therefore focus the discussion on the mitochondrial III_2_IV_1/2_ supercomplexes. A discussion of a functional significance of these mitochondrial supercomplexes is complicated by the variability in their composition, the variable distribution of free complexes and supercomplexes in different organisms,^54,278^ and the differences in relative orientation of the respiratory complexes within the supercomplexes, i.e., the flexibility in the interaction surfaces of the supercomplex components among different species (Figure 7). Nevertheless, it is well established that supercomplexes do form in a wide range of organisms and are likely to have functional significance. As already indicated above, various physiological roles of supercomplexes have been discussed (e.g., refs (23,53−55,279,280)), and below we summarize some specific suggestions with a focus on cyt. *bc*_1_–Cyt*c*O supercomplexes.

### 6.1 Changes in Structure or Activity upon Formation of Supercomplexes

The lack of well-defined structural changes of the respiratory enzymes upon association into supercomplexes, and the differences in the relative orientation of the components in different organisms (Figure 7) suggest that formation of supercomplexes does not result in changes in functionality of individual components. Changes in turnover activity of individual respiratory complexes upon forming supercomplexes have been reported, but they are typically too small to yield any functionally relevant changes in the overall electron flux through the respiratory chain (see refs (53,54)). Furthermore, as outlined above, the electron flux through the respiratory chain *in vivo* is typically lower than the *k*_cat_ values of the components. Therefore, formation–dissociation of the mitochondrial supercomplexes is unlikely to comprise a universal mechanism to modulate function through changes of the activity of complexes III or IV themselves.

A similar problem is associated with identifying specific effects of supercomplex formation on the “stability” of the components, which has been suggested in the past, although mainly for complex I (reviewed in refs (53,54)). As pointed out by Milenkovic *et al*.,^53^ many of the studies addressing this issue are based on observation of correlations of effects on function, structure and morphology, and it is at present not possible to deduce any specific mechanistic effects at a molecular level.

### 6.2 Protein Distribution and Aggregation

Blaza *et al*.^281^ proposed that formation of supercomplexes is a consequence of the very high protein density of the inner mitochondrial membrane (∼2/3 protein); formation of supercomplexes would outcompete irreversible, unspecific aggregation of respiratory complexes with other membrane components.^53,281^ However, as also noted by these authors, in mammalian mitochondria only 15–30% of Cyt*c*O is part of supercomplexes.^54^ This equilibrium of free complexes and supercomplexes indicates that association of respiratory complexes to form supercomplexes is realized through relatively weak reversible interactions. Because a reversible equilibrium of supercomplexes and free complexes could not block irreversible formation of aggregates between respiratory complexes and other membrane proteins, we consider this role of supercomplexes to be less likely.

In *S. cerevisiae*, a larger fraction (∼90%) of the Cyt*c*O population is part of supercomplexes.^72^ An equilibrium constant between supercomplex-bound and free Cyt*c*O in the order of 10 suggests that also in *S. cerevisiae*, the III_2_IV_1/2_ supercomplexes are held together by weak interactions. This conclusion is further supported by the necessity to use weak detergents for isolation of supercomplexes (e.g., digitonin or glyco-diosgenin, GDN) and the observation that they dissociate into components upon addition of *n*-dodecyl-β-d-maltoside (DDM).^37^ Thus, also in *S. cerevisiae* the cyt. *bc*_1_–Cyt*c*O interactions are reversible and could not outcompete irreversible nonspecific aggregation with other membrane-bound proteins.

Another suggestion for the role of supercomplexes originates from an observation of the preference for respiratory complexes for specific membrane topology.^282^ Fedor and Hirst^283^ suggested that formation of supercomplexes would ensure an even distribution of the respiratory complexes in the membrane, a plausible proposal that could be tested experimentally in future studies.

### 6.3 Production of ROS

Formation of supercomplexes has been suggested to decrease the amount of produced reactive oxygen species (ROS) (e.g., refs (73,284)). Here, we briefly discuss this proposed role in the framework of effects at a molecular level. This discussion requires a definition of the term ROS as it does not describe a single chemical entity, but rather a range of molecules or ions that are formed upon incomplete reduction of O_2_ (i.e., reduction by <4 electrons), including superoxide, peroxide, and hydroxyl radicals.^285^ The reactivity of these species differs and therefore the term ROS only depicts a generally reactive molecule or ion. Reduction of O_2_ by one electron at a time yields first the superoxide anion (O_2_^•–^), which is the precursor of other ROS.^285,286^ The main sites of initial O_2_^•–^ formation in mitochondria are at complexes I and III.^285,286^

The amount formed O_2_^•–^ at a specific redox site at a particular O_2_ concentration is determined by the relative rates of O_2_^•–^ formation (“side reaction”) and the rate by which the electron is transferred from that site to the next acceptor in the electron-transfer chain (physiological reaction). When assuming that formation of supercomplexes would yield less ROS, the implicit assumption is that the electron-transfer rate away from the ROS-forming site would be slower for individually diffusing complexes than for supercomplexes.

Data from studies of model systems suggest that the amount of ROS at complex I decreases upon supercomplex formation.^284^ However, Fedor and Hirst^283^ recently showed that QH_2_ produced by complex I in supercomplexes is oxidized to Q more rapidly outside the supercomplex than by the acceptor within the supercomplex (complex III). In other words, electrons from complex I are removed more rapidly in the absence than in the presence of supercomplexes. As a consequence, formation of supercomplexes that involve complex I would not *per se* result in decreasing the fraction of reduced ROS-forming sites at complex I.

A postulate that formation of supercomplexes composed of cyt. *bc*_1_ and Cyt*c*O would yield less ROS implies that association of the components would result in a faster reoxidation of cyt. *bc*_1_ because ROS is mainly formed at cyt. *bc*_1_. Indeed, as discussed above, reduction–oxidation of cyt. *c* is the rate-limiting step of electron transfer from QH_2_ (complex III) to O_2_ (complex IV) in *S. cerevisiae*. Therefore, a decrease in this transfer rate upon dissociation of the III_2_IV_1/2_ supercomplexes would result in a larger fraction of reduced complex III, which could result in accumulation of electrons at the Q_P_ site where nonphysiological reduction of O_2_ to O_2_^•–^ is most likely to take place.^81^ Hence, we consider it possible that O_2_^•^^–^ production is indeed lowered upon formation of III_2_IV_1/2_ supercomplexes.

In the above discussion, we consider a fully functional respiratory chain. However, in the native membrane, new respiratory complexes are continuously produced, and at a given time there are also partly assembled respiratory complexes with incompletely connected electron-transfer chains. These partly assembled complexes could accumulate electrons at their redox sites, which upon interaction with O_2_ may form ROS. It is possible that association of these partly assembled complexes with other fully functional partner complexes to form supercomplexes^287^ would provide a route for dissipation of these reducing equivalents. In so doing, the probability for ROS formation from partly assembled respiratory complexes would be diminished.

### 6.4 Free Energy Conservation

As already discussed above, early hypotheses suggesting “substrate channeling”, i.e., direct transfer of confined Q/QH_2_ or cyt. *c* between respiratory complexes within a supercomplex, have been rejected.^38,53,88,269,281,283^ Yet, supercomplexes have been proposed to allow a “more efficient” transport of electrons and an increase in the “efficiency” of respiration allowing higher “yields” of energy conservation (see e.g., refs (25,36,56,76,282,288)). Therefore, a consideration of effects of supercomplex formation on “efficiency” and “yield”, terms frequently used in the discussions, requires a definition of these terms and a more detailed analysis.

The free energy available at each respiratory complex (energy input) is defined by the difference in standard redox potentials of the electron donor and acceptor, the concentration ratio of reduced and oxidized donor, as well as the concentration ratio of reduced and oxidized acceptor. The free energy conserved at each respiratory complex (energy output) is determined by the number of protons transferred across the membrane and the charge separation upon oxidation of the electron donor and reduction of the acceptor. The term efficiency typically depicts the ratio of free energy output and free energy input in a given system. An assumption that association of respiratory–chain complexes into supercomplexes results in an increased efficiency of respiration implies that the efficiency of at least one component would increase. However, as discussed above, changes in the charge-separation stoichiometry of individual complexes are unlikely to occur upon association into supercomplexes and therefore the overall efficiency of the system is not expected to change upon forming supercomplexes.

The terms “yield” and “efficiency” are in principle equivalent but are often used in different context. The former is often used to depict the amount of ATP formed for a given amount of oxidized substrate of the respiratory chain (cf., the so-called P/O ratio). This parameter is also determined by the efficiency of each component, including the ATP synthase and, hence, it is not expected to change upon association of respiratory complexes into supercomplexes.

It is relevant to note that the yield of ATP formation is also dependent on proton leaks across the membrane. Proton leaks often occur at protein–membrane interface surfaces, which become smaller upon association of respiratory complexes into supercomplexes. However, the protein–protein interaction surface upon formation of a supercomplex comprises only a very small fraction of the sum of all protein–membrane interaction surfaces of all membrane proteins of the inner mitochondrial membrane. Therefore, the effect of decreasing the protein–membrane interaction surface upon forming supercomplexes would most likely not result in increasing the yield of ATP production. That said, it is clear that an intricate web of regulatory pathways in mitochondria controls energy conservation in respiratory complexes and the overall P/O ratio, depending on environmental conditions.^289^ These regulatory pathways may also involve formation and dissociation of supercomplexes. However, changes in the energy-conversion efficiency or yield cannot simply be a direct consequence of changing the distance between respiratory complexes to form supercomplexes.

If “more efficient” incorrectly alludes to an increase in the electron-transfer rate between respiratory complexes, the suggestion that supercomplex formation would result in “more efficient” electron transfer is plausible, at least when considering association of complexes III and IV (see above).

### 6.5 The Redox State and Binding of cyt. *c*

We consider electron transfer between complexes III and IV via cyt. *c* diffusion and discuss two scenarios: (*i*) freely diffusing complexes III and IV where after reduction at cyt. *bc*_1_, cyt. *c* equilibrates with the cyt. *c* pool in the intermembrane space and electrons are transferred to Cyt*c*O from this cyt. *c* pool (Figure 10A); (*ii*) electron transfer from cyt. *bc*_1_ to Cyt*c*O by 2D diffusion along the surface of a CIII_2_CIV_1/2_ supercomplex (Figure 10B). According to scenario (*i*), the redox state of the cyt. *c* pool in the intermembrane space is determined by the relative rates of cyt. *c* reduction at cyt. *bc*_1_ and oxidation at Cyt*c*O. According to scenario (*ii*), the redox state of the cyt. *c* pool is determined by the equilibrium constant of cyt. *c* bound to the supercomplex surface and free cyt. *c* in the bulk solution, i.e., the probability that a surface-associated cyt. *c* in the reduced state is replaced by a bulk oxidized cyt. *c*. In addition, cyt. *c* from the cyt. *c* pool may transiently interact and exchange electrons with any of the complexes or the bound cyt. *c* during the 2D electron transfer. Nevertheless, the reduction level of the cyt. *c* pool is expected to depend on the fractions cyt. *bc*_1_ and Cyt*c*O that are part of a supercomplex because the nature of the electronic link changes upon supercomplex formation/dissociation. As proposed by Moe *et al*.,^37^ the scenario suggests yet another possible functional role of supercomplex formation, i.e., to alter the reduced:oxidized ratio of cyt. *c*. Because cyt. *c* is involved in an intricate web of cellular interactions,^290,291^ there may be a link between assembly of cyt. *bc*_1_ and Cyt*c*O into supercomplexes, changes in environmental conditions, and cellular redox-signaling pathways.

Yet another possibility is that formation of supramolecular assemblies is not directly linked to functional properties of the respiratory chain. Cytochrome *c* is a positively charged dipolar molecule, which resides in an environment containing negatively charged proteins.^247^ Association of cyt. *c* with the supercomplex surface by electrostatic interactions may be necessary to outcompete nonspecific reversible binding to other negatively charged proteins and membrane surfaces in the intermembrane space. Formation of supercomplexes that allow electron transfer by 2D diffusion along the supercomplex surface could thus be a consequence of the electrostatic binding of cyt. *c* to cyt. *bc*_1_ and Cyt*c*O.

The discussion above leaves us with a question: why do mitochondria use a soluble, diffusible cyt. *c* rather than a membrane-anchored counterpart? In this context, it is interesting to recapitulate that *R. prowazekii*, the closest known microbe relative of mitochondria,^257,259^ harbors only a membrane-anchored cyt. *c*_y_ homologue.^257,259^ We speculate that if the role of cyt. *c* is only to shuttle electrons between cyt. *bc*_1_ and Cyt*c*O, then at a minimal cyt. *c* concentration, the highest possible electron-transfer rate is maintained by a membrane-anchored cyt. *c*. However, evolution has given also other, regulatory functions to cyt. *c*, such as, e.g., being a messenger in apoptosis,^203,291^ which is linked to the redox properties of this electron carrier and may require a water-soluble, diffusible variant. A “best of both worlds” scenario, e.g., in *S. cerevisiae*, would therefore be to keep the same electron-transfer mechanism as that in *R. prowazekii* by association of cyt. *c* with a cyt. *bc*_1_–Cyt*c*O supercomplex surface, but to use a water-soluble cyt. *c* that can also sustain other mitochondrial functions.

## 7 Final Remarks

Respiratory supercomplexes are found in a wide range of organisms. Structures of the bacterial and mitochondrial III_2_IV_1/2_ supercomplexes show a great variability in their overall composition and relative orientations of the components, which suggests that the only common structural characteristics of the supramolecular assemblies is proximity of the components. Cryo-EM structures of the III_2_IV_1/2_ supercomplexes show that the components are connected via a small number of protein–protein interactions as well as interfacial cardiolipin, and the structures of cyt. *bc*_1_ and Cyt*c*O remain essentially unaltered upon association. Collectively, the data suggest that the functional role of the supramolecular assemblies is to minimize the distance between the components. We suggest that this organization supports a mechanism that allows electron transfer by 2D diffusion of cyt. *c* across the merged negatively charged surface of the supercomplex.^37^ The consequence of electron transfer by 2D diffusion upon forming a supercomplex is a change in the fraction of reduced/oxidized cyt. *c* in the intermembrane space, which may be sensed by multiple regulatory pathways of the cell. Alternatively, the 2D diffusion mechanism may be a consequence of tight binding of cyt. *c* to cyt. *bc*_1_ and Cyt*c*O in order to outcompete nonspecific interactions between cyt. *c* and negatively charged proteins and membrane surfaces in the intermembrane space. In actinobacteria, electron transfer from complex III to complex IV is conducted via the diheme cyt. *cc* domain of subunit QcrC. In these supercomplexes, there is an additional effect from the intricate intertwining and shared structural domains, which suggests that the supercomplex functions as a single unit. This unit also comprises novel key structural features such as an FeS domain that is locked at a fixed position in complex III and a complex III “lid” that shapes a novel proton pathway orifice in complex IV. Future studies will hopefully reveal the functional significance of these novel structural features and offer further general insights into the functional significance of respiratory supercomplexes at a molecular level.
